# Kinase PLK1 regulates the disassembly of the lateral elements and the assembly of the inner centromere during the diakinesis/metaphase I transition in male mouse meiosis

**DOI:** 10.3389/fcell.2022.1069946

**Published:** 2023-01-13

**Authors:** Rocío Gómez, Alberto Viera, Tania Moreno-Mármol, Inés Berenguer, Andrea Guajardo-Grence, Attila Tóth, María Teresa Parra, José A. Suja

**Affiliations:** ^1^ Unidad de Biología Celular, Departamento de Biología, Facultad de Ciencias, Universidad Autónoma de Madrid, Madrid, Spain; ^2^ Departamento de Neuropatología Molecular, Centro de Biología Molecular Severo Ochoa, Campus de la Universidad Autónoma de Madrid, Madrid, Spain; ^3^ Hospital Universitario Santa Cristina, Instituto de Investigación Sanitaria del Hospital Universitario de La Princesa, Universidad Autónoma de Madrid, Madrid, Spain; ^4^ Institute of Physiological Chemistry, Faculty of Medicine, Technische Universität Dresden, Dresden, Germany

**Keywords:** mouse, meiosis, PLK1, lateral elements, inner centromere, H2AT120ph, H3T3ph

## Abstract

PLK1 is a serine/threonine kinase with crucial roles during mitosis. However, its involvement during mammalian male meiosis remains largely unexplored. By inhibiting the kinase activity of PLK1 using BI 2536 on organotypic cultures of seminiferous tubules, we found that the disassembly of SYCP3 and HORMAD1 from the lateral elements of the synaptonemal complex during diakinesis is impeded. We also found that the normal recruitment of SYCP3 and HORMAD1 to the inner centromere in prometaphase I spermatocytes did not occur. Additionally, we analyzed the participation of PLK1 in the assembly of the inner centromere by studying its implication in the Bub1-H2AT120ph-dependent recruitment of shugoshin SGO2, and the Haspin-H3T3ph-dependent recruitment of Aurora B/C and Borealin. Our results indicated that both pathways are regulated by PLK1. Altogether, our results demonstrate that PLK1 is a master regulator of the late prophase I/metaphase I transition in mouse spermatocytes.

## Introduction

Meiosis is a specialized cell division process characterized by a single round of DNA replication followed by two rounds of chromosome segregation, which promotes the generation of haploid gametes. During prophase of the first meiotic division (prophase I), the homologous chromosomes must correctly achieve their pairing, synapsis and recombination to allow a successful chromosome segregation during the first meiotic division (Handel and Schimenti, 2010; Bolcun-Filas and Handel, 2018). These processes lead to the formation of a meiosis-specific zipper-like proteinaceous structure known as the synaptonemal complex (SC), the hallmark of meiosis (Fraune et al., 2012; Zhang et al., 2021). The SC is formed by two lateral elements (LEs), one per homolog, and a series of transverse filaments connecting them. The transverse filaments interact at the SC central region forming the central element (CE) (Fraune et al., 2012). During the leptotene stage of prophase I the so-called axial elements (AEs) form along each homolog and are then named LEs once the homologs begin to pair during the zygotene stage. Mammalian AEs/LEs are mainly composed of the proteins SYCP2 and SYCP3 (Moens et al., 1987; Dobson et al., 1994; Schalk et al., 1998), different cohesin complexes (Suja and Barbero, 2009; McNicoll et al., 2013), the cohesin regulatory proteins NIPBL and MAU2 (Visnes et al., 2014), condensin complexes (Visnes et al., 2014), and the recruited HORMA-domain proteins HORMAD1 and HORMAD2 (Wojtasz et al., 2009). During pachytene, the homologs are synapsed and SCs are fully formed along the length of the autosomal bivalents. Once recombination is completed, the homologs and their LEs desynapse by diplotene due to the disassembly of CE proteins (Jordan et al., 2012). Studies on mouse spermatocytes indicated a gradual disassembly of the LE protein SYCP3 during late prophase I stages, and its accumulation at metaphase I inner centromeres (Dobson et al., 1994; Prieto et al., 2001; Eijpe et al., 2003; Parra et al., 2004; Gómez et al., 2007). However, the precise sequence of events leading to these processes, and their regulation, are poorly understood in vertebrates (Cahoon and Hawley, 2016; Gao and Colaiácovo, 2018; Láscarez-Lagunas et al., 2022).

Different studies have pointed out to potential kinases that would be responsible for the SC and LE disassembly. In budding yeast meiosis, the kinases Cdc5/PLK1, Ipl1/Aurora B, Ddk and Cdk play important roles (Clyne et al., 2003; Sourirajan and Lichten, 2008; Jordan et al., 2009; Argunhan et al., 2017). In male mouse meiosis, the Polo-like kinase PLK1 also promotes SC disassembly (Ishiguro et al., 2011). PLK1 phosphorylates the CE proteins SYCP1 and TEX12 to allow desynapsis of homolog LEs (Jordan et al., 2012). Interestingly, it has been recently reported that the kinases Aurora B and C, as well as PLK1, regulate the disassembly of LEs during the late prophase I/metaphase I transition (Wellard et al., 2020; Wellard et al., 2022).

PLKs are a family of serine/threonine kinases conserved from yeast to mammals (Korns et al., 2022). There are several PLK paralogs in mammals, PLK1-5, but PLK1 is the most studied one. Many publications have shown that during mammalian mitotic and meiotic divisions PLK1 is localized at the centrosomes, acentriolar microtubule organizing centres (MTOCs), kinetochores, the central spindle and the mid-body. Accordingly, PLK1 is a key regulator of mitosis and female mouse meiosis since it has key roles in mitotic entry and meiotic resumption, formation of acentriolar MTOCs, centrosome maturation and separation, bipolar spindle assembly, kinetochore-microtubule attachment, chromosome condensation, alignment and segregation, regulation of the anaphase-promoting complex/cyclosome (APC/C), and cytokinesis (Tong et al., 2002; Schmucker and Sumara, 2014; Kim et al., 2015; Solc et al., 2015; Combes et al., 2017; Little and Jordan, 2020). In contrast, the role of PLK1 in male meiosis is much less understood in comparison with female meiosis.

By phosphorylating some cohesin subunits PLK1 is also responsible for the partial release of cohesin complexes from chromosome arms during vertebrate mitotic prophase and prometaphase, the so-called “prophase pathway” (Giménez-Abián et al., 2004; Hauf et al., 2005). In addition, it has been proposed that in mammalian somatic cells PLK1 phosphorylates and activates the kinase Haspin (Ghenoiu et al., 2013; Zhou et al., 2014). Haspin then phosphorylates histone H3 at threonine 3 (H3T3ph) (Dai et al., 2005) creating a platform for the recruitment of the kinase Aurora B and other chromosomal passenger complex (CPC) proteins to the inner centromere (Kelly et al., 2010; Wang et al., 2010; Yamagishi et al., 2010; Wang et al., 2011; De Antoni et al., 2012; Wang et al., 2012).

Here, we analyzed the participation of PLK1 in the disassembly of the SC LEs and the REC8 cohesin axes of chromosomes during the diakinesis/metaphase I transition. We first studied the accurate pattern of distribution of the LE proteins SYCP3 and HORMAD1, and of the REC8-containing cohesin axes, during the diakinesis/metaphase I transition in wild-type (WT) spermatocytes. Then, we inhibited the kinase activity of PLK1 by treating organotypic cultures of seminiferous tubules with BI 2536, a small potent molecule that specifically inhibits PLK1 in somatic cells (Lénárt et al., 2007; Steegmaier et al., 2007; Zhou et al., 2014; Su et al., 2022), and mouse spermatocytes (Alfaro et al., 2021) and oocytes (Pomerantz et al., 2012; Du et al., 2015; Kim et al., 2015; Solc et al., 2015). Moreover, we also analyzed the putative participation of PLK1 in the H2AT120ph- and H3T3ph-dependent recruitment of the inner centromere proteins SGO2 and the CPC proteins Aurora B/C and Borealin, respectively. Our results show that PLK1 is needed for the disassembly of LEs during the diakinesis/metaphase I transition, and the loading of SGO2 and CPC proteins to the inner centromeres during the first meiotic division in mouse spermatocytes.

## Results

### SYCP3 and HORMAD1 disassemble similarly from LEs during the diakinesis/metaphase I transition

**FIGURE 1:**
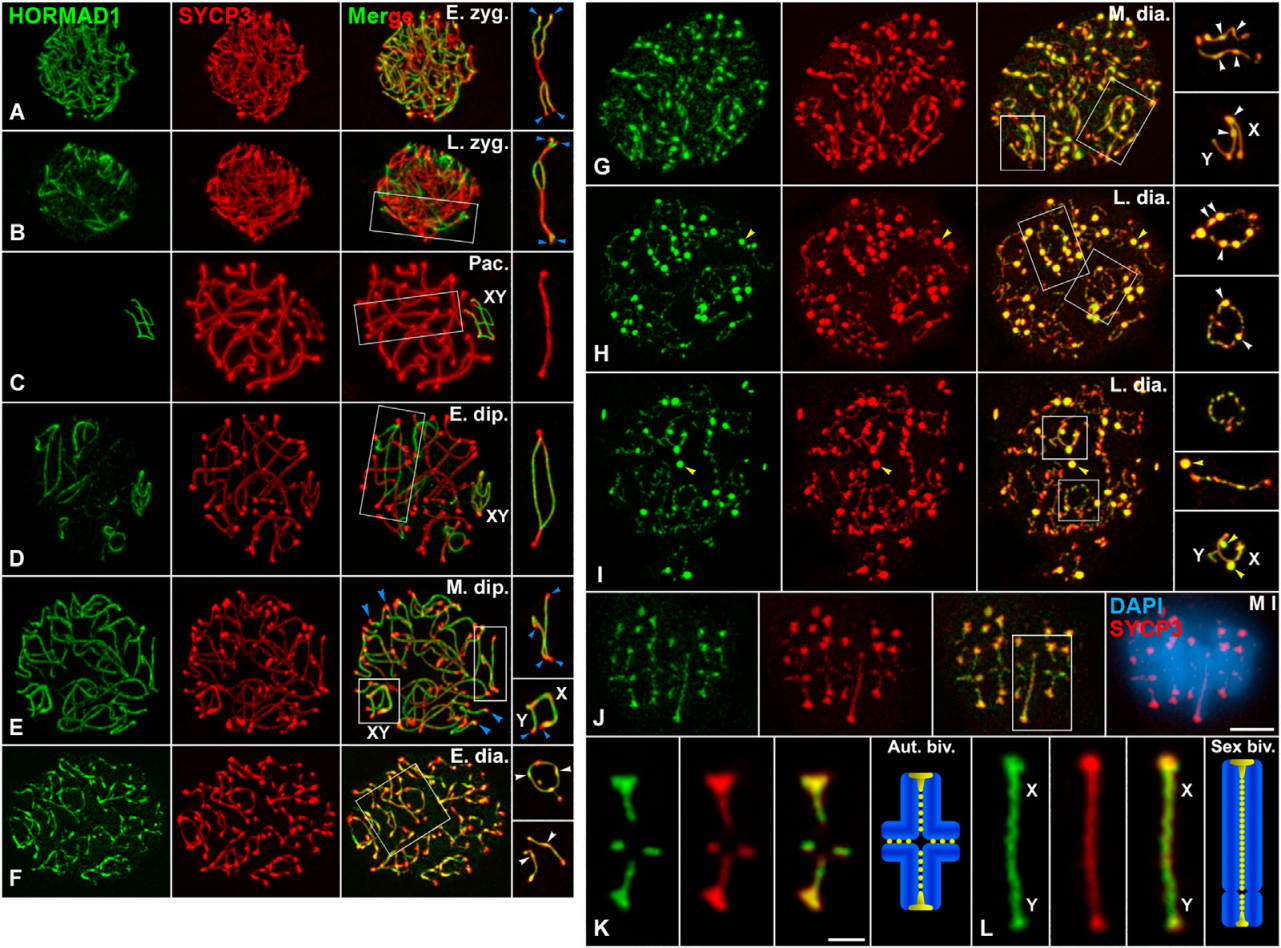
HORMAD1 and SYCP3 are similarly released from the LEs during the diakinesis/metaphase I transition. Double immunolabeling of HORMAD1 (green) and SYCP3 (red), and counterstaining of the chromatin with DAPI (blue) on squashed WT spermatocytes. Representative spermatocytes at **(A)** early zygotene (E. zyg.), **(B)** late zygotene (L. zyg.), **(C)** pachytene (Pac.), **(D)** early diplotene (E. dip.), **(E)** mid diplotene (M. dip.), **(F)** early diakinesis (E. dia.), **(G)** mid diakinesis (M. dia.), **(H,I)** late diakinesis (L. dia.), and **(J)** metaphase I (M I) spermatocytes are shown. **(K,L)** Selected metaphase I autosomal (Aut. biv.) **(K)** and sex (Sex biv.) **(L)** bivalents. The sex body (XY) is indicated if recognizable. Selected autosomal and sex (XY) bivalents in squared regions are shown in the right column **(A–J)** and the lower right line **(K,L)**. Blue arrowheads indicate telomere regions with absence of HORMAD1 labeling. White arrowheads indicate elongated bulges and round thickenings of HORMAD1 and SYCP3 along desynapsed autosomal LEs, and asynapsed AEs of the X chromosome. Yellow arrowheads indicate HORMAD1 and SYCP3 agglomerates in the nucleoplasm of late diakinesis spermatocytes **(H,I)**. Scale bars represent 5 μm in **(A–J)**, and 2 μm in **(K,L)**.

Since one of our main goals was to determine the potential role of PLK1 in the disassembly of the LEs during the diakinesis/metaphase I transition, a meiotic window poorly characterized in males, we first analyzed the accurate and “step-by-step” dynamics of SYCP3 and HORMAD1 during this transition in WT spermatocytes. For this purpose, we made a double immunolabeling of these proteins on squashed spermatocytes. We used the squashing technique because it doesn’t disturb nuclear volume and integrity, and chromosome condensation and distribution in prophase I nuclei and dividing spermatocytes are preserved (Page et al., 1998; Parra et al., 2002). In fact, we used this technique previously to describe a concise distribution of SYCP3 (Parra et al., 2004; Parra et al., 2006). Our present results showed, as previously described (Wojtasz et al., 2009), that HORMAD1 and SYCP3 colocalized along the asynapsed AEs in zygotene spermatocytes (Figures 1A,B), and that during the pachytene stage HORMAD1 preferentially labeled the asynapsed AEs of the sex chromosomes (Figure 1C). During the diplotene stage, HORMAD1 and SYCP3 colocalized again along the desynapsed LEs except at their ends, that were only labeled by SYCP3, in both autosomal and sex bivalents (Figures 1D,E; Supplementary Video S1). Interestingly, in early diakinesis spermatocytes both proteins colocalized not only along the desynapsed LEs, with some of their stretches becoming thinner at this stage, but also at elongated bulges that began to appear along them (Figure 1F; Supplementary Video S1). In diakinesis spermatocytes, SYCP3 also appeared as a homogeneous and intense nuclear background, a hallmark of all diakinesis substages, when observed by the squashing technique. Since about 75–85 focal planes were captured for each diakinesis nucleus, and Z-projections in a single plane obscured the SYCP3 labeling along the LEs, we substracted this background with the ImageJ software for improving clarity (Supplementary Figure S1). Shortly afterwards, in mid diakinesis spermatocytes, HORMAD1 and SYCP3 also colocalized along the thin desynapsed LEs and at numerous round thickenings along them (Figure 1G). The colocalization and distribution patterns of these proteins were likewise present in late diakinesis spermatocytes (Figures 1H,I). However, at this stage, in addition to the round thickenings along the LEs, which began to appear discontinuous, both proteins colocalized at some large round agglomerates that didn’t localize at LEs or centromeres, and apparently were in the nucleoplasm (Figures 1H,I). In order to precisely determine the staging of diakinesis spermatocytes and avoid a confusion with prometaphases I and metaphases I, we made a double immunolabeling of SYCP3 and lamin B, to indirectly reveal the integrity of the nuclear envelope. Our results showed the presence of a continuous nuclear envelope even in late diakinesis spermatocytes, and its initial disintegration in prometaphase I spermatocytes (Supplementary Figures S2A–E). A double immunolabeling of SYCP3 and the inner nuclear membrane protein SUN1, that associates to the telomeres, showed that even in late diakinesis spermatocytes the ends of desynapsed LEs appeared attached to the nuclear envelope (Supplementary Figures S2F–H).

In metaphase I autosomal bivalents, HORMAD1 and SYCP3 also colocalized at small patches present along the region of contact between sister-chromatid arms, previously named the interchromatid domain (Suja et al., 1999; Prieto et al., 2001; Parra et al., 2004) (Figures 1J,K; Supplementary Figure S3). The labeling of both proteins was more continuous at the interchromatid domain of the sex bivalent (Figure 1L, Supplementary Figure S3). Moreover, both proteins also appeared highly accumulated at the centromeres (Figures 1J,K; Supplementary Figure S3). A double immunolabeling of SYCP3 or HORMAD1 and the kinetochores, revealed by an ACA serum, showed that they were accumulated at the inner centromere domain below the associated sister kinetochores (Supplementary Figures S4, S5). It is worth noting that at this stage both proteins also appeared at large round agglomerates in the cytoplasm like those observed in the nucleoplasm of late diakinesis nuclei (Supplementary Figures S2E, S3, S5E). We also analyzed the distribution of SYCP3 during the diplotene/metaphase I transition with the spreading technique, that is the procedure commonly used in male mouse meiosis studies. Our results showed that diakinesis spermatocytes were scarce and difficult to find, but the dynamics of SYCP3 was like that observed on squashed spermatocytes at autosomal and sex bivalents (Supplementary Figures S6, S7). However, bulges and thickenings along desynapsed LEs, as well as nucleoplasmic agglomerates in spread diakinesis nuclei were difficult to observe, probably due to the spreading procedure. Altogether, our results indicate that SYCP3 and HORMAD1 are released similarly from the LEs during the diakinesis/metaphase I transition, to then accumulate preferentially at the inner centromeres in metaphase I chromosomes.

### SYCP3 and REC8-containing cohesin complexes are differentially released during the diakinesis/metaphase I transition

**FIGURE 2:**
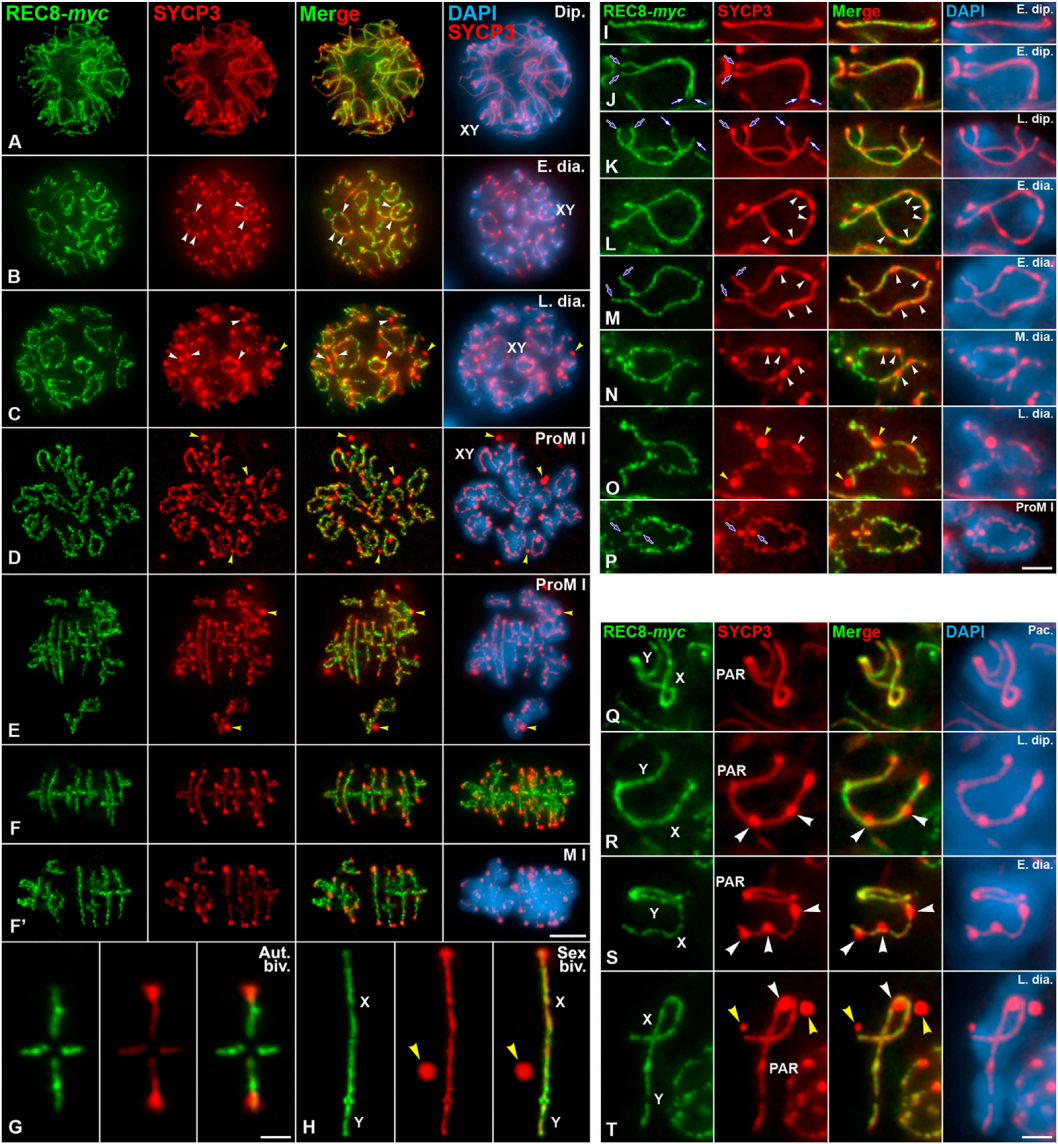
Dynamics of REC8 and SYCP3 are distinct during the diakinesis/metaphase I transition. Double immunolabeling of REC8-*myc* (green) and SYCP3 (red), and counterstaining of the chromatin with DAPI (blue) on squashed WT spermatocytes. Representative spermatocytes at **(A)** diplotene (Dip.), **(B)** early diakinesis (E. dia.), **(C)** late diakinesis (L. dia.), **(D,E)** prometaphase I (ProM I), and **(F,F’)** metaphase I (M I) are shown. **(G,H)** Selected metaphase I autosomal (Aut. biv.) **(G)** and sex (Sex biv.) **(H)** bivalents. The sex body and the sex bivalents (XY) are indicated in prophase I and prometaphase I spermatocytes. White arrowheads indicate elongated bulges and round SYCP3 thickenings along the desynapsed LEs in early **(B)** and late **(C)** diakinesis. Yellow arrowheads indicate SYCP3 agglomerates in the nucleoplasm of late diakinesis **(C)**, and in the cytoplasm of prometaphase I **(D,E)** and metaphase I **(H)** spermatocytes. Selected autosomal bivalents are shown at **(I,J)** early diplotene (E. dip.), **(K)** late diplotene (L. dip.), **(L,M)** early diakinesis (E. dia.), **(N)** mid diakinesis (M. dia.), **(O)** late diakinesis (L. dia.), and **(P)** prometaphase I (ProM I). Blue and white arrows indicate the proximal centromeric and distal telomere regions, respectively. White arrowheads indicate elongated bulges and round SYCP3 thickenings along the desynapsed LEs in early **(L,M)**, mid **(N)**, and late **(O)** diakinesis. Yellow arrowheads indicate SYCP3 agglomerates in the nucleoplasm of a late diakinesis **(O)** spermatocyte. Selected sex bivalents are shown at **(Q)** pachytene (Pac.), **(R)** late diplotene (L. dip.), **(S)** early diakinesis (E. dia.), and **(T)** late diakinesis (L. dia.). The X and Y chromosomes, and the PAR region, are indicated. White arrowheads indicate round SYCP3 thickenings along the asynapsed AE of the X chromosome in late diplotene **(R)**, and early and late diakinesis **(S,T)**. Yellow arrowheads indicate SYCP3 agglomerates in the nucleoplasm of a late diakinesis spermatocyte **(T)**. Scale bars represent 5 μm in **(A–F’)**, 1 μm in **(G,H)**, 2 μm in **(I–P)**, and 1 μm in **(Q–T)**.

We also aimed to ascertain the potential role of PLK1 in the partial disassembly of the meiotic cohesin axes during the diakinesis/metaphase I transition. To this end, and although the pattern of localization of the meiotic cohesin subunit REC8 has been previously reported in mouse spermatocytes (Eijpe et al., 2003; Lee et al., 2003; Kudo et al., 2006), we first analyzed in detail its dynamics and compared it with that of SYCP3. For this, we codetected REC8, in *myc* tagged version of REC8 mice (REC8-*myc*) (Kudo et al., 2006) and SYCP3 on squashed spermatocytes. REC8 and SYCP3 axes colocalized in their trajectories from leptotene up to diplotene (Supplementary Figure S8; Figure 2A). By contrast, in early and late diakinesis spermatocytes REC8 appeared as discontinuous lines at the cohesin axes (Figures 2B,C). In prometaphase I and metaphase I spermatocytes, REC8 and SYCP3 decorated similarly the interchromatid domain of autosomal and sex bivalents (Figures 2D–F′). Nevertheless, REC8 didn’t colocalized with SYCP3 at the cytoplasmic agglomerates and at the inner centromeres (Figures 2F–H). An accurate analysis of the dynamics of REC8 and SYCP3 on autosomal and sex bivalents corroborated that these proteins had different behaviors during the late prophase I/metaphase I transition (Figures 2I–T). The labeling of REC8 at cohesin axes, which underlie the autosomal LEs and the asynapsed sex chromosomes AEs, became discontinuous from late diplotene/early diakinesis on (Figures 2K–P, R–T). These results indicate that a partial release of REC8-containing cohesin complexes along cohesin axes occurs during these stages. By contrast, the labeling of SYCP3 became discontinuous along autosomal LEs from mid diakinesis on, concomitantly with the appearance of thickenings along them (Figures 2N–P). Altogether, our results point that SYCP3 and REC8 are differentially released from the desynapsed autosomal LEs and cohesin axes, respectively, during diakinesis, and that REC8 doesn’t accumulate at the whole inner centromeres in prometaphase I and metaphase I chromosomes (Figures 2G,H; Supplementary Figure S9).

### *In vitro* inhibition of PLK1 kinase activity in organotypic cultures of seminiferous tubules

**FIGURE 3:**
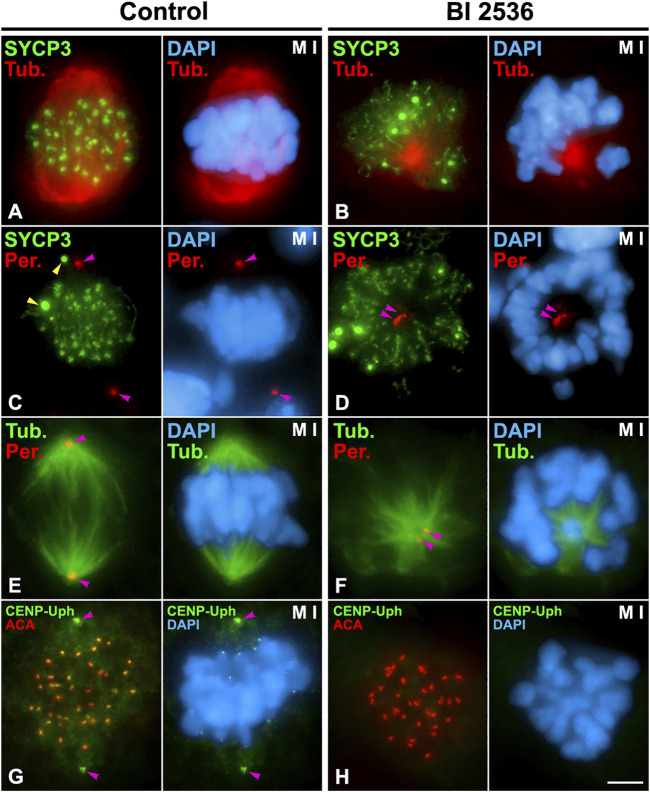
Efficiency of BI 2536 on cultured seminiferous tubules. Double immunolabelings of SYCP3 (green) and α-Tubulin (red) **(A,B)**; SYCP3 (green) and Pericentrin (red) **(C,D)**; α-Tubulin (green) and Pericentrin (red) **(E,F)**; or CENP-Uph (phosphorylated at T78) (green) and kinetochores (ACA, red) **(G,H)**, and counterstaining of the chromatin with DAPI (blue) on squashed control **(A,C,E,G)** and 8 h BI 2536-treated **(B,D,F,H)** metaphase I spermatocytes. Yellow arrowheads indicate SYCP3 agglomerates in the cytoplasm of a control metaphase I spermatocyte **(C)**. Pink arrowheads indicate the centrosomes **(C,D,E,F,G)**. Scale bar represents 5 μm.

In order to determine the role of PLK1 in the disassembly of the LEs we inhibited its kinase activity *in vitro* with the pharmacological inhibitor BI 2536 on organotypic cultures of seminiferous tubules, as previously reported (Jordan et al., 2012; Alfaro et al., 2021). In a previous study we tested different concentrations of BI 2536 on cultured seminiferous tubules to inhibit the kinase activity of PLK1 without affecting the viability of cultured spermatocytes (Alfaro et al., 2021). We decided to use a concentration of 100 μM BI 2536 and 8 h of treatment since with these conditions low levels of apoptosis were found as detected with Caspase 3 (Alfaro et al., 2021), and a TUNEL assay on squashed control non-inhibited spermatocytes (3.3% of apoptotic spermatocytes, n = 1,000) and inhibited spermatocytes (5.40% of apoptotic spermatocytes, *n* = 1,000) (Supplementary Figure S10). We confirmed the efficiency of the inhibition in three different individuals by detecting, after double immunolabeling of α-Tubulin and Pericentrin, that 55,36% of metaphases I (*n* = 466) were altered and showed unaligned bivalents, monopolar spindles and unseparated centrosomes, as previously reported (Alfaro et al., 2021; Wellard et al., 2021) (Figures 3A–F). In this regard, this kind of altered metaphases I was never observed in control non-inhibited spermatocytes (*n* = 500). Moreover, we determined that in all altered metaphases I (*n* = 30) the phosphorylation of CENP-U at its threonine 78, a phosphorylation introduced by PLK1 (Kang et al., 2006), wasn’t detected at centrosomes or kinetochores (Figures 3G,H).

**FIGURE 4:**
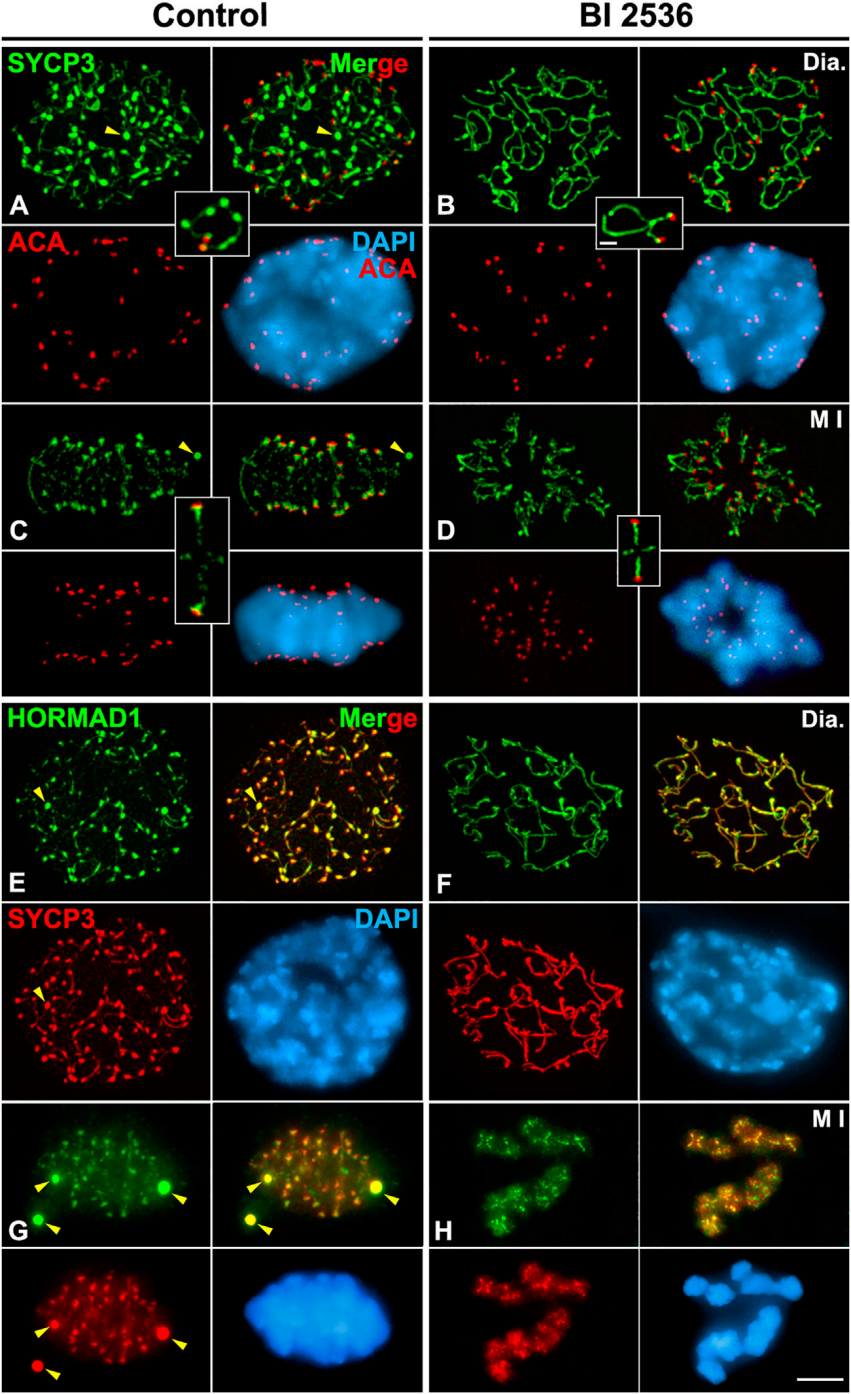
PLK1 regulates the release of SYCP3 and HORMAD1 from desynapsed LEs in diakinesis and its accumulation at the inner centromeres in metaphase I spermatocytes. Double immunolabelings of SYCP3 (green) and kinetochores (ACA, red) **(A–D)**, and SYCP3 (red) and HORMAD1 (green) **(E–H)**, and counterstaining of the chromatin with DAPI (blue) on squashed control **(A,C,E,G)** and BI 2536-treated **(B,D,F,H)** spermatocytes. Representative spermatocytes and selected autosomal bivalents at **(A,B,E,F)** diakinesis (Dia.), and **(C,D,G,H)** metaphase I (M I) are shown. Yellow arrowheads indicate SYCP3 agglomerates in the nucleoplasm of diakinesis spermatocytes **(A,E)**, and in the cytoplasm of metaphase I **(C,G)** spermatocytes. Scale bars represent 5 μm in **(A–H)**, and 1 μm in selected diakinesis and metaphase I bivalents in **(A–D)**.

In all control 8 h cultured diakinesis spermatocytes (*n* = 25), as in WT spermatocytes, thin SYCP3-labelled LEs with thickenings along them were observed (Figure 4A). By contrast, with an 8 h BI 2536 treatment we found that in all diakinesis spermatocytes (*n* = 30) the labeling of SYCP3 was more continuous along desynapsed LEs, no thickenings were detected along them, and nucleoplasmic agglomerates were never observed (Figure 4B; Supplementary Video S2). After an 8 h treatment, altered metaphases I with unaligned bivalents, always (*n* = 235) presented an intense and continuous labeling of SYCP3 at the interchromatid domain of autosomal and sex bivalents (Figures 4C,D; Supplementary Video S3). Concomitantly, we found that in those altered metaphases I SYCP3 wasn’t accumulated at the inner centromere of the chromosomes, albeit the close association of sister kinetochores hadn’t changed (Figures 4C,D; Supplementary Video S3). Interestingly, no anaphase I or telophase I spermatocytes were detected in 8 h BI 2536-treated seminiferous tubules indicating that altered metaphases I remained arrested and didn’t progress in the division process. This contrasted with the situation found in control seminiferous tubules were anaphases I and telophases I were always observed. The characteristic and recognizable SYCP3 labelling displayed by altered monopolar metaphases I, being continuous at the interchromatid domain but absent at the inner centromere (Figures 3B,D, 4C,D **)**, was employed in the rest of our analyses to identify metaphases I altered by the *in vitro* inhibition of PLK1 kinase activity.

### PLK1 regulates the dynamics of HORMAD1 and SYCP3 during the diakinesis/metaphase I transition

We analyzed the putative role of PLK1 in the disassembly of desynapsed LEs during the diakinesis/metaphase I transition. First, we double immunolabeled HORMAD1 and SYCP3 on 8 h BI 2536-cultured seminiferous tubules. We found that in all altered diakinesis spermatocytes both proteins colocalized as continuous lines decorating the desynapsed LEs without thickenings along them, a completely different appearance in relation to that found in control spermatocytes (Figures 4E,F; Supplementary Video S4). Similarly, in all altered metaphase I bivalents HORMAD1 and SYCP3 colocalized at their interchromatid domain showing an intense labeling along them (Figures 4G,H; Supplementary Video S5). Interestingly, both proteins were not enriched at the inner centromeres, contrasting with the labeling observed in control spermatocytes (Figures 4G,H; Supplementary Video S5). These results indicate that the kinase activity of PLK1 is necessary for the regular disassembly of HORMAD1 and SYCP3 from the desynapsed LEs and their subsequent accumulation at the inner centromeres.

### Inhibition of PLK1 has no apparent direct effect on REC8, RAD21, and RAD21L distributions during the diakinesis/metaphase I transition

**FIGURE 5:**
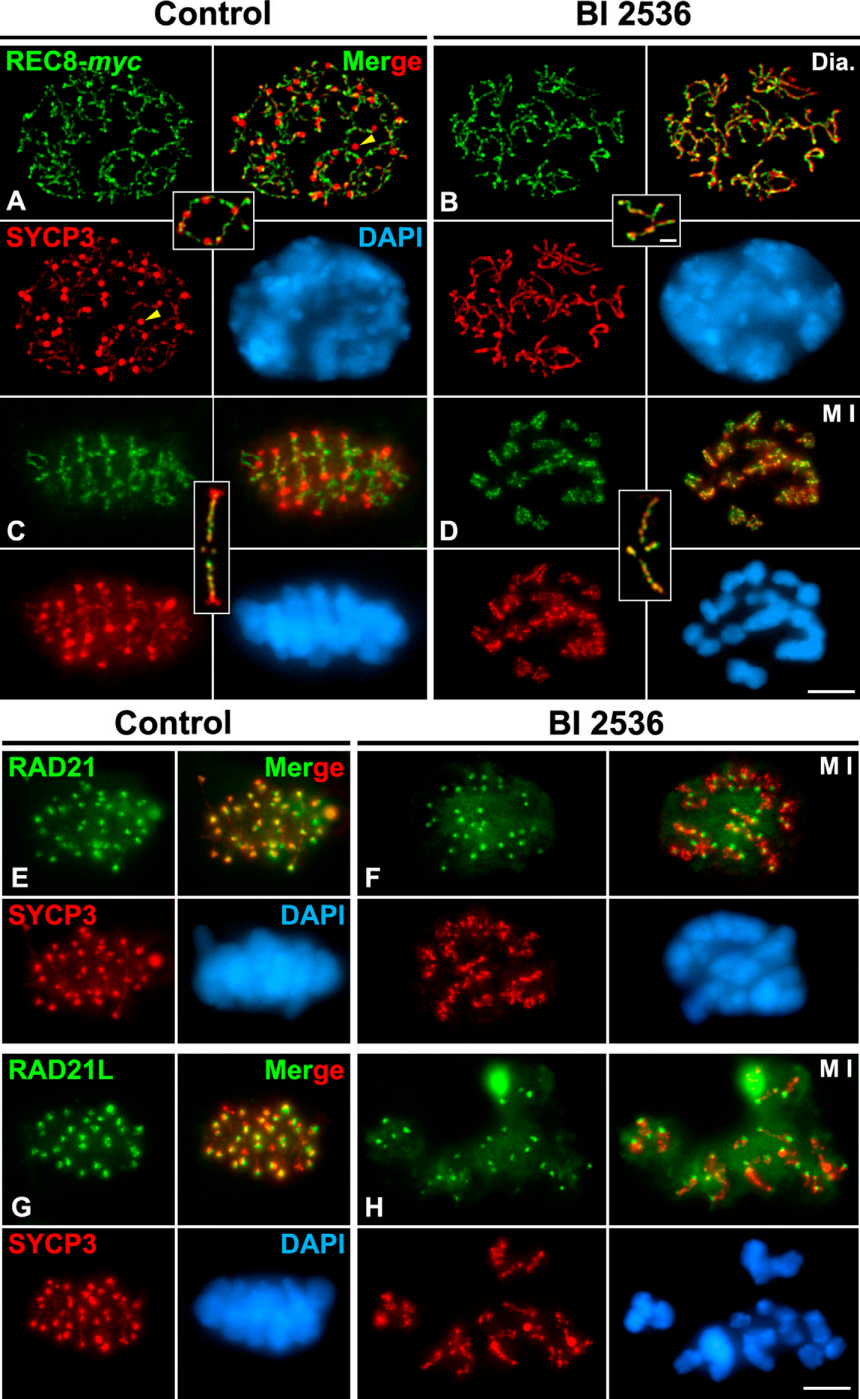
Inhibition of PLK1 does not alter the distribution of REC8, RAD21 and RAD21L-contanining cohesin complexes in diakinesis and metaphase I spermatocytes. Double immunolabelings of REC8-*myc* (green in **A–D**), RAD21 (green in **E,F**) or RAD21L (green in **G,H**) and SYCP3 (red), and counterstaining of the chromatin with DAPI (blue) on squashed control **(A,C,E,G)** and BI 2536-treated **(B,D,F,H)** diakinesis (Dia.) **(A,B)** and metaphase I (M I) spermatocytes **(C–G)**. Selected autosomal bivalents in diakinesis **(A,B)** and metaphase I **(C,D)** spermatocytes are shown. Yellow arrowheads indicate agglomerates of SYCP3 in the nucleoplasm of the control diakinesis spermatocyte. Scale bars represent 5 μm in **(A–H)**, and 1 μm in selected diakinesis and metaphase I bivalents in **(A–D)**.

We next examined the behavior of REC8 on BI 2536-treated altered diakinesis and metaphase I spermatocytes. The double labeling of REC-*myc* and SYCP3 demonstrated that the distribution of REC8 along cohesin axes on altered diakinesis spermatocytes was like that found in control ones (Figures 5A,B; Supplementary Video S6). REC8 appeared as a discontinuous labeling along the cohesin axes (Figures 5A,B). On the other hand, in altered metaphase I bivalents REC8 was found as a series of bright patches along the interchromatid domain of chromosome arms that slightly penetrated the inner centromeres, as in control bivalents (Figures 5C,D; Supplementary Video S7). We also analyzed whether the inhibition of PLK1 could affect the distributions of RAD21-and RAD21L-containing cohesin complexes in metaphase I spermatocytes. It has been reported that RAD21 (Parra et al., 2004; Gómez et al., 2007; Viera et al., 2007) and RAD21L (Herrán et al., 2011; Ishiguro et al., 2011) appear highly accumulated at the inner centromere of WT metaphase I chromosomes, in contrast to REC8 distribution (Suja and Barbero, 2009). Our results showed that RAD21 (Figures 5E,F) and RAD21L (Figures 5G,H) showed the same distribution at the inner centromeres in both control and BI 2536-treated metaphase I chromosomes. Altogether, our results indicate that the kinase activity of PLK1 is needed for the regular disassembly and redistribution of HORMAD1 and SYCP3, but it apparently doesn’t affect the distribution of REC8, RAD21 or RAD21L-containing cohesin complexes.

### PLK1 regulates H2AT120ph phosphorylation and the loading of shugoshin SGO2 and MCAK to the inner centromere

**FIGURE 6:**
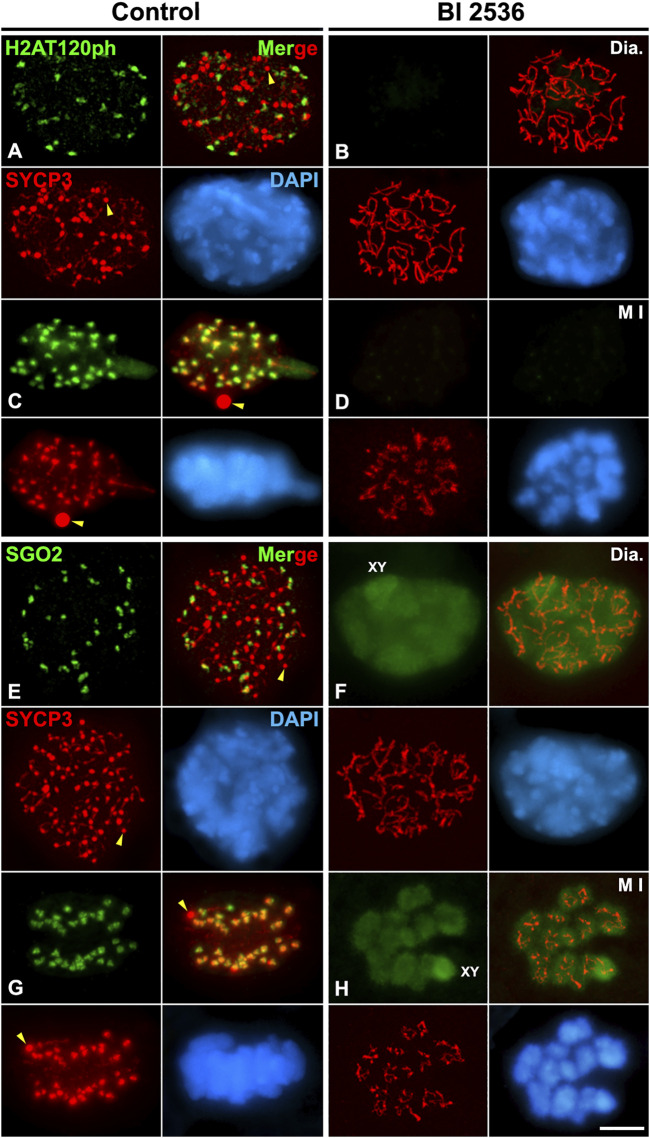
PLK1 regulates the H2AT120ph-dependent loading of SGO2 to the inner centromeres. Double immunolabelings of SYCP3 (red) with either H2AT120ph (green in **A–D**), or SGO2 (green in **E–H**), and counterstaining of the chromatin with DAPI (blue) on squashed diakinesis (Dia.) **(A,E)** and metaphase I (M I) **(C,G)** control spermatocytes, and diakinesis **(B,F)** and metaphase I **(D,H)** BI 2536-treated spermatocytes. The sex bivalent (XY) is indicated in **(F,H)**. Yellow arrowheads indicate SYCP3 agglomerates in the nucleoplasm and cytoplasm of control diakinesis and metaphase I spermatocytes, respectively. Scale bar represents 5 μm.

**FIGURE 7:**
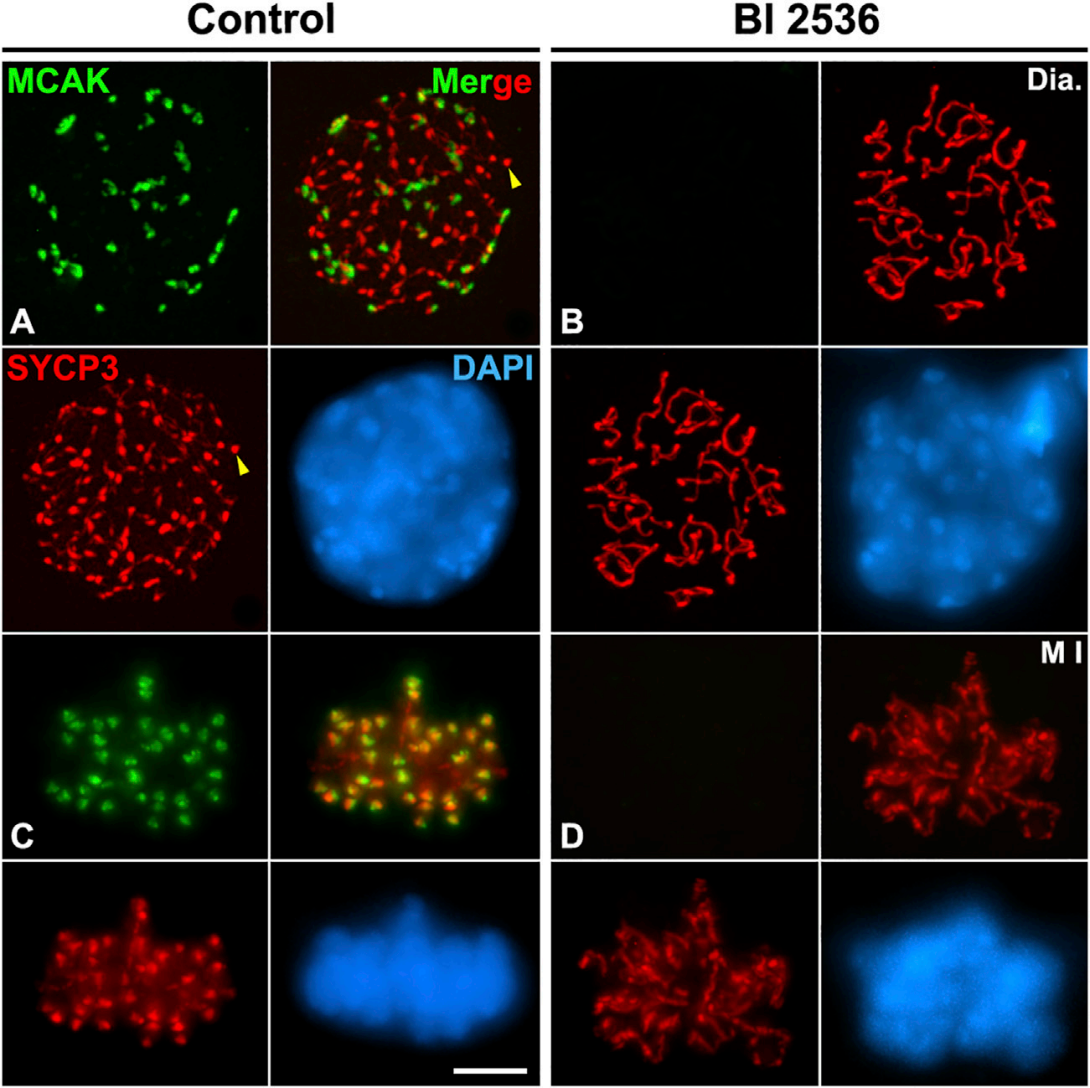
PLK1 regulates the SGO2-dependent loading of MCAK to the inner centromeres. Double immunolabeling of MCAK (green) and SYCP3 (red), and counterstaining of the chromatin with DAPI (blue) on squashed diakinesis (Dia.) **(A)** and metaphase I (M I) **(C)** control spermatocytes, and diakinesis **(B)** and metaphase I **(D)** BI 2536-treated spermatocytes. Yellow arrowheads indicate SYCP3 agglomerates in the nucleoplasm of the control diakinesis spermatocyte. Scale bar represents 5 μm.

Since we observed that HORMAD1 and SYCP3 weren’t loaded to the inner centromeres in altered metaphase I bivalents, we also tested a potential PLK1 function in the loading of other proteins that normally load to the inner centromere. For this purpose, we studied the two main pathways that regulate the assembly of the inner centromere domain: the pathway Bub1-H2AT120ph-Shugoshin SGO2, and the pathway Haspin-H3T3ph-Aurora B. The phosphorylation of histone H2A at threonine 120 by the kinase Bub1 is necessary to recruit the cohesin protector protein Shugoshin SGO1 to the centromeres (Jeganathan et al., 2007; Kawashima et al., 2010; Wang and Higgins, 2012; Watanabe, 2012). The histone modification H2AT120ph was not detected at the centromeres in altered diakinesis and metaphase I spermatocytes (Figures 6A–D). Accordingly, we corroborated that without the centromere presence of H2AT120ph, SGO2 wasn’t detected at the inner centromeres in altered diakinesis and metaphase I spermatocytes. Nevertheless, SGO2 appeared dispersed over the chromatin in diakinesis nuclei and metaphase I bivalents, particularly on the sex bivalent (Figures 6E–H). In addition, since it has been reported that SGO2 recruits the microtubule depolymerizing kinesin MCAK to the inner centromeres in mouse spermatocytes and oocytes (Llano et al., 2008; Tanno et al., 2010; Rattani et al., 2013), we compared the distribution of MCAK in control and BI 2536-treated diakinesis and metaphase I spermatocytes. We found that MCAK wasn’t recruited at the inner centromere in altered diakinesis and metaphase I spermatocytes (Figures 7A–D). Our results thus indicate that PLK1 regulates the phosphorylation of H2AT120ph at the centromeres, and the subsequent loading of SGO2 and MCAK to the inner centromeres in diakinesis and metaphase I bivalents.

### PLK1 regulates the phosphorylations of H3T3ph and Aurora B/C, and the loading of Borealin at the inner centromere

**FIGURE 8:**
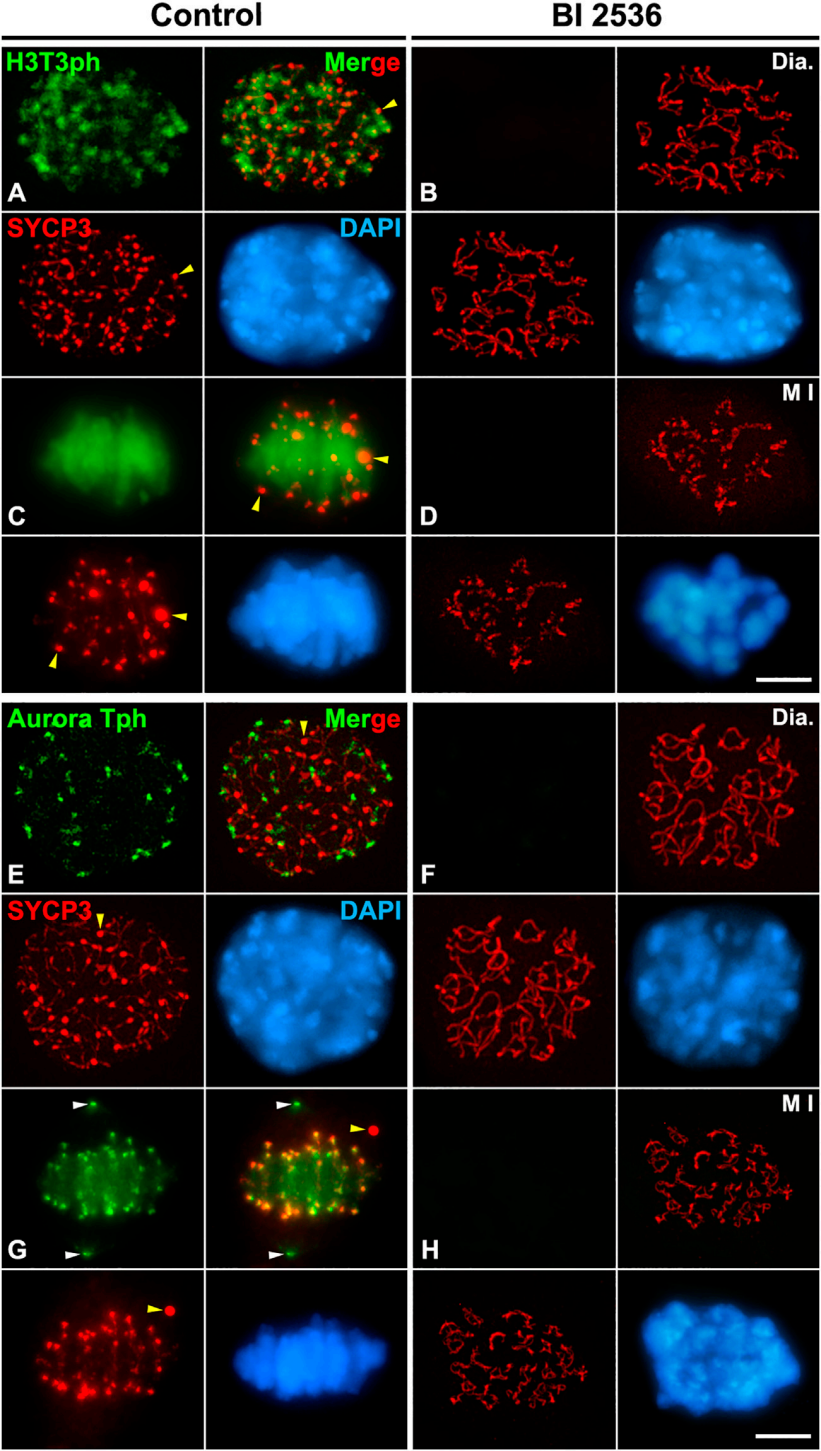
PLK1 regulates the H3T3ph-dependent phosphorylation of Aurora B/C at the inner centromeres. Double immunolabelings of SYCP3 (red) with either H3T3ph (green in **A–D**) or AuroraTph (green in **E–H**), and counterstaining of the chromatin with DAPI (blue) on squashed diakinesis (Dia.) **(A,E)** and metaphase I (M I) **(C,G)** control spermatocytes, and diakinesis **(B,F)** and metaphase I **(D,H)** BI 2536-treated spermatocytes. Yellow arrowheads indicate SYCP3 agglomerates in the nucleoplasm and cytoplasm of control diakinesis and metaphase I spermatocytes, respectively. White arrowheads in **(G)** indicate the centrosomes. Scale bar represents 5 μm.

**FIGURE 9:**
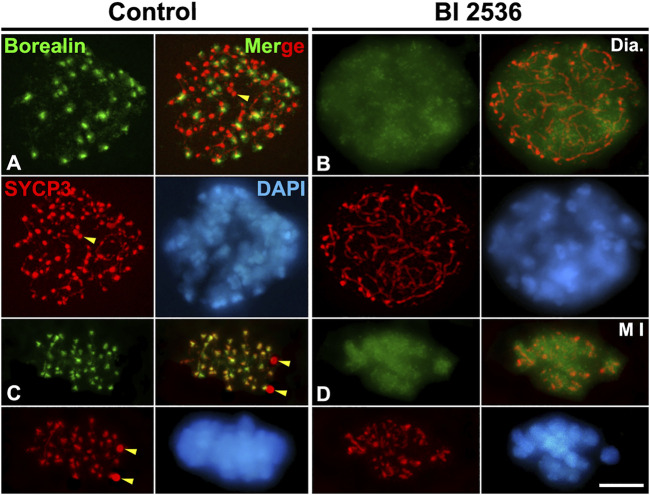
PLK1 regulates the loading of Borealin to the inner centromeres. Double immunolabeling of Borealin (green) and SYCP3 (red), and counterstaining of the chromatin with DAPI (blue) on squashed diakinesis (Dia.) **(A)** and metaphase I (M I) **(C)** control spermatocytes, and diakinesis **(B)** and metaphase I **(D)** BI 2536-treated spermatocytes. Yellow arrowheads indicate SYCP3 agglomerates in the nucleoplasm and cytoplasm of the control diakinesis and metaphase I spermatocytes, respectively. Scale bar represents 5 μm.

We next tested whether PLK1 regulates the Haspin-H3T3ph-dependent loading of Aurora B/C to the inner centromere during the diakinesis/metaphase I transition. We found that H3T3ph was present at chromocenters, which represent clustered centromeres, in control diakinesis spermatocytes, and covering the chromatin in control metaphase I bivalents (Figures 8A,C). However, H3T3ph was undetectable in altered diakinesis or metaphase I spermatocytes (Figures 8B,D). Then, we analyzed the distribution of Aurora B/C at the centromeres by using an antibody that recognizes phosphorylated forms of Aurora A, B, and C, an antibody herein called Aurora Tph. With this antibody we detected a labeling at the centromeres in control diakinesis spermatocytes, as previously reported (Parra et al., 2003; Parra et al., 2009) (Figure 8E). In control metaphase I, we observed a labeling at the centrosomes, which corresponds to the labeling of Aurora A (Willems et al., 2018; Alfaro et al., 2021; Berenguer et al., 2022), and at the inner centromeres, which corresponds to the labeling of the kinases Aurora B/C (Watanabe, 2010; Balboula and Schindler, 2014; Hindriksen et al., 2017; Alfaro et al., 2021; Berenguer et al., 2022) (Figure 8G). In contrast, no labeling was observed in altered diakinesis and metaphase I spermatocytes (Figures 8F,H). We also analyzed whether the loading of the CPC protein Borealin was disturbed after inhibiting PLK1. Our results showed that Borealin appeared at the inner centromeres in diakinesis and metaphase I control spermatocytes (Figures 9A,C), butn’t in altered diakinesis and metaphase I spermatocytes (Figures 9B,D). Altogether these results indicate that PLK1 regulates the phosphorylations of H3T3ph and Aurora B/C, and the loading of Borealin at the inner centromeres during male mouse meiosis I.

### Phosphorylated forms of PLK1 localize at LEs and inner centromeres

Our results indicated that PLK1 regulated the disassembly of HORMAD1 and SYCP3 from the LEs, the phosphorylations of H2AT120ph, H3T3ph and Aurora B/C, and the loading of SGO2, MCAK, and Borealin at the inner centromeres during the diakinesis/metaphase I transition. In order to study whether PLK1 could be present at SCs and inner centromeres, we studied the distribution of PLK1 phosphorylated at serine 137 (PLK1S137ph) and at threonine 210 (PLK1T210ph) in spread spermatocytes. We found that PLK1S137ph appeared at the centrosomes and on the AEs/LEs from leptotene up to the diakinesis stage (Supplementary Figures S11A–J). Interestingly, PLK1S137ph colocalized with SYCP3 at the bulges and thickenings present along the asynapsed AEs of the sex chromosomes in late diplotene spermatocytes (Supplementary Figures S11G–I), and along the desynapsed LEs in diakinesis spermatocytes (Supplementary Figure S11J). On the other hand, PLK1S137ph appeared accumulated at the inner centromeres of prometaphase I and metaphase I bivalents (Supplementary Figures S11K,L). Differentially, PLK1T210ph didn’t appear at the centrosomes, and was only detected at the centromeres from diplotene stage on, and was observed enriched at the inner centromere domain, colocalizing with SYCP3, in diakinesis, prometaphase I, and metaphase I spermatocytes (Supplementary Figure S12). These results indicate that different posttranslational modifications of PLK1 are at the right place to presumably mediate either in the disassembly of LEs and/or the assembly of the inner centromere in mouse spermatocytes.

## Discussion

### Dynamics of LEs disassembly during the diakinesis/metaphase I transition

**FIGURE 10:**
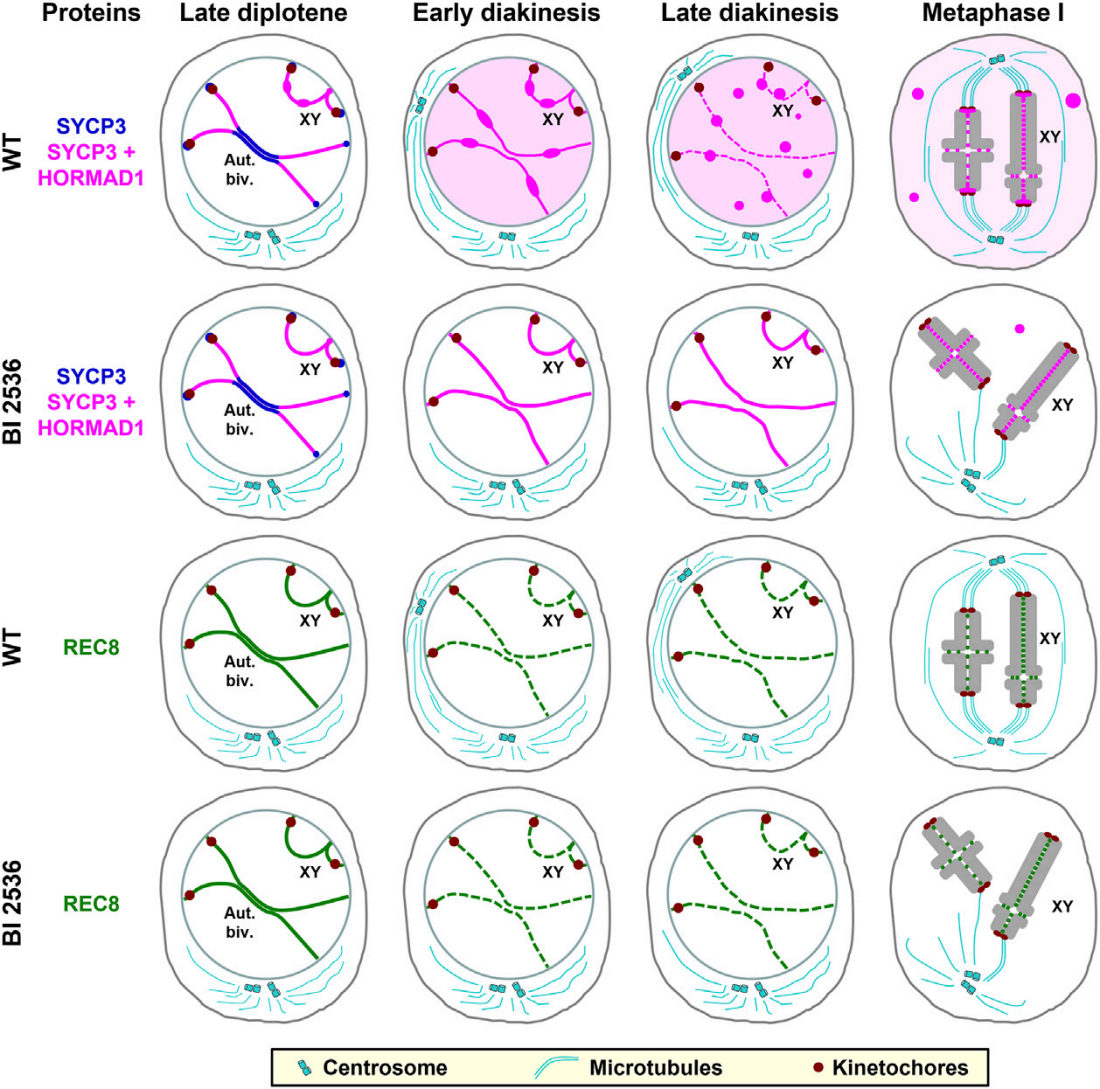
Schematic representation of the distribution of SYCP3, HORMAD1 and REC8 in WT and BI 2536-treated late diplotene, early and late diakinesis, and metaphase I spermatocytes. For clarity, a single autosomal bivalent (Aut. Biv.) and the sex bivalent (XY) are represented. SYCP3 is indicated in blue, the merge of SYCP3 and HORMAD1 in pink, and REC8 in green. Bulges and round thickenings along desynapsed LEs, as well as nucleoplasmic or cytoplasmic agglomerates of SYCP3 and HORMAD1, are indicated. A nucleoplasmic and cytoplasmic background of SYCP3 and HORMAD1 are indicated as a pale pink background in WT late diakinesis and metaphase I spermatocytes. Kinetochore regions are shown in brown, and centrioles and microtubules in light blue. The condensed chromatin of the autosomal bivalent, with a single interstitial chiasma, and of the sex bivalent (XY), with a distal chiasma, is shown in light grey in metaphase I spermatocytes.

In previous reports a concise description of the distribution of SYCP3 and HORMAD1 in diakinesis and metaphase I spermatocytes was presented (Parra et al., 2004; Parra et al., 2006; Wojtasz et al., 2009). In this sense, SYCP3 disassembles from the LEs during late prophase I stages and then accumulate at the centromeres in metaphase I spermatocytes (Cahoon and Hawley, 2016; Gao and Colaiácovo, 2018; Láscarez-Lagunas et al., 2022). However, a precise description of this dynamic hasn’t been reported. Here we have analyzed, for the first time, the accurate behavior of SYCP3 and HORMAD1 during the diakinesis/metaphase I transition in WT mouse spermatocytes. Our results show that both proteins have the same pattern of distribution and behavior during this transition. Thus, in early diakinesis spermatocytes the HORMAD1-and SYCP3-labeled desynapsed LEs become thinner, and frequent bulges along them appear concomitantly with an increase of the nuclear background. In mid diakinesis spermatocytes, round thickenings along the LEs are observed, while in late diakinesis spermatocytes LEs become discontinuous and nucleoplasmic agglomerates appear. Remnants of HORMAD1 and SYCP3 are detected at the interchromatid domain, and are preferentially accumulated at the inner centromere domain, of prometaphase I and metaphase I bivalents. This observed sequence of events is summarized in Figure 10. Considering these data, we propose a working model for the disassembly of the LEs during the diakinesis/metaphase I transition. SYCP3 and HORMAD1, which interact between them and form complexes (Fukuda et al., 2010; Fujiwara et al., 2020), could begin to be released from desynapsed autosomal LEs at early diakinesis. During this stage, one population of these complexes could accumulate at bulges along LEs, while another population could diffuse in the nucleoplasm. With the ongoing release of these proteins in mid and late diakinesis, there could be a concentration of these proteins on previous bulges to appear as larger round thickenings along the LEs, that in turn become discontinuous. We hypothesize that some SYCP3/HORMAD1 complexes present at those thickenings could diffuse to aggregate as nucleoplasmic agglomerates. Alternatively, among other possibilities, the thickenings could detach as agglomerates from LEs to directly lie in the nucleoplasm. The formation of agglomerates in the nucleoplasm is supported by the fact that SYCP3 self-assembles in the nucleoplasm and cytoplasm when expressed in cultured somatic cells (Yuan et al., 1998). However, a population of SYCP3 and HORMAD1 still persists as small patches at the interchromatid domain in prometaphase I and metaphase I bivalents. Another interesting question is how those proteins accumulate at the inner centromere in prometaphase I bivalents. In this regard, newly synthesized proteins or non-degraded proteins that previously diffused from the LEs to the nucleoplasm or were present at nucleoplasmic agglomerates could be recruited to the inner centromeres. Obviously, more reserch is needed to evaluate these or other possibilities. We consider that high-resolution observations on living spermatocytes expressing SYCP3 and/or HORMAD1 tagged with GFP during the diakinesis/metaphase I transition would allow a better definition of the observed steps of LEs disassembly. In addition, FRAP experiments would offer outstanding information on the rate of synthesis and behavior of these proteins during this transition.

Concerning the dynamics of REC8-containing cohesin axes during the diakinesis/metaphase I transition, our results point that these cohesin complexes would initiate their release from desynapsed LEs earlier that SYCP3 and HORMAD1 do. In this sense, the partial release of those cohesin complexes could lead to the discontinuity of cohesin axes from early diakinesis on. Interestingly, released cohesin complexes weren’t cytologically detected in the nuclear background. Thus, we suggest that the released cohesin complexes, probably not cleaved by Separase, could be degraded as it seems to occur during the mitotic “prophase pathway” (Giménez-Abián et al., 2004; Hauf et al., 2005). Other REC8-containing cohesin complexes would be protected against their release from chromosome arms during diakinesis to persist at the interchromatid domain of metaphase I bivalents to ensure sister-chromatid arm and centromere cohesion until anaphase I segregation. In summary, REC8-containing cohesin complexes, and SYCP3 and HORMAD1 are differentially released from cohesin axes and desynapsed LEs during the diakinesis/metaphase I transition, probably by still non-characterized and different molecular mechanisms.

### PLK1 regulates the disassembly of LEs during the diakinesis/metaphase I transition

Previous experiments on *in vitro* cultures of mouse spermatocytes with the PLK1 inhibitor GW843682X, and posterior induction to undergo metaphase I with the addition of okadaic acid, promoted the retention of SYCP3 along the arms of metaphase I bivalents (Ishiguro et al., 2011). Accordingly, these authors proposed that PLK1 might promote the release of SYCP3 during late prophase I stages. In addition, it has been recently observed that in *Plk1* cKO male mice the disassembly of the LEs is aberrant (Wellard et al., 2022). In this sense, the LE proteins SYCP3 and SYCP2 were retained at the interchromatid domain of metaphase I bivalents, and these proteins were absent at the centromeres. These results led the authors to propose that PLK1 is required for LEs disassembly (Wellard et al., 2022). We have found that after the *in vitro* inhibition of the kinase activity of PLK1 with BI 2536 altered diakinesis spermatocytes show a continuous labeling of SYCP3 and HORMAD1 along the desynapsed LEs. Remarkably, in these spermatocytes neither thickenings along them nor nucleoplasmic agglomerates are found. Moreover, altered metaphases I, that probably enter the first meiotic division as altered diakinesis during the duration of the BI 2536 treatment, show a continuous labeling of these proteins at the interchromatid domain of bivalents, and don’t accumulate at their inner centromeres. These data suggest that the SYCP3 and HORMAD1 proteins that normally accumulate at the inner centromeres in prometaphase I bivalents derive from the population of proteins that are previously released from the LEs throughout the diakinesis stage. Altogether, our results strongly support previous results (Ishiguro et al., 2011; Wellard et al., 2022) indicating that PLK1 regulates the disassembly of the LEs during the diakinesis/metaphase I transition by enabling the release not only of SYCP3, but also of HORMAD1. Since during budding yeast meiosis Cdc5/PLK1 also controls this disassembly (Sourirajan and Lichten, 2008; Argunhan et al., 2017), we suggest that PLK1 could be considered as a master kinase that controls this meiotic process.

It is worth noting that we have found that PLK1 phosphorylated at serine S137 (PLK1S137ph) is present along the AEs/LEs during all prophase I stages, and at bulges and thickenings along desynapsed LEs in late diplotene and diakinesis spermatocytes. The phosphorylation of PLK1S137ph is necessary to activate PLK1 to phosphorylate some targets in the S interphase stage (Jang et al., 2002), and has been detected in mouse oocytes (Du et al., 2015; Feitosa et al., 2018) and spermatocytes (Wellard et al., 2022). Our results suggest that this active phosphorylated form of PLK1 is at the right place to mediate the phosphorylation of the LE proteins leading to their release.

Our results show that the distribution of REC8 at desynapsed cohesin axes during diakinesis, and at the interchromatid domain of metaphase I bivalents, are similar in altered, control and WT spermatocytes. These results apparently suggest that PLK1 would not be required for the partial release of REC8-complexes during the diakinesis/metaphase I transition. However, it must be considered that desynapsed REC8 axes appeared discontinuous at early diakinesis. Thus, with our experimental conditions, i.e., an 8 h BI 2536 treatment, we cannot disregard the possibility that PLK1, by promoting the phosphorylation of REC8 or other subunits of those complexes during late diplotene, could promote the partial release of REC8 from cohesin axes. This early release of REC8 cohesin complexes during late diplotene/early diakinesis could allow the posterior PLK1-dependent release of SYCP3 and HORMAD1 from LEs, as previously suggested (Ishiguro et al., 2011). It has been proposed that the cohesin regulator WAPL could allow the dissociation of cohesin complexes from the cohesin axes in prophase I mouse spermatocytes and oocytes (Brieño-Enríquez et al., 2016; Silva et al., 2020). On the other hand, it has been reported that during the so-called “prophase I-like pathway” in budding yeast meiosis there is a cleavage-independent release of Rec8 cohesin complexes from the SC during late prophase I promoted by Cdc5/PLK1, Rad61/Wpl1/WAPL, and the Dbf4-dependent Cdc7 kinase (DDK) in a collaborative way (Challa et al., 2019a; Challa et al., 2019b).

On the other hand, our results on the distribution of the cohesin subunits RAD21 and RAD21L in control and BI 2536-altered metaphases I showed that their distributions at the centromeres weren’t affected. These results suggest that their accumulation at the inner centromeres in metaphase I spermatocytes, as previously reported (Parra et al., 2004; Gómez et al., 2007; Viera et al., 2007; Herrán et al., 2011; Ishiguro et al., 2011), aren’t dependent of PLK1.

### PLK1 regulates the loading of inner centromere proteins

There are two main pathways that regulate the assembly of the inner centromere domain: the pathway Bub1-H2AT120ph-Shugoshin SGO2, and the pathway Haspin-H3T3ph-Aurora B (Watanabe, 2010; Yamagishi et al., 2010; Hadders et al., 2020; Schmitz et al., 2020). In the first pathway, the kinase Bub1 phosphorylates histone H2A at threonine 120, which then recruits the centromere cohesin protector protein Shugoshin SGO1/2 in somatic cells, and mouse oocytes and spermatocytes (Jeganathan et al., 2007; Kawashima et al., 2010; Ricke et al., 2012; Wang and Higgins, 2012; Watanabe, 2012; El Yakoubi et al., 2017). In the second pathway, the kinase Haspin phosphorylates histone H3 at threonine 3 (H3T3ph) (Dai et al., 2005), which then recruits the kinase Aurora B to the inner centromere (Kelly et al., 2010; Wang et al., 2010; Yamagishi et al., 2010; Wang et al., 2011; De Antoni et al., 2012; Wang et al., 2012). Our results indicate that in the absence of PLK1 kinase activity H2AT120ph isn’t phosphorylated, and SGO2 isn’t loaded to the centromeres in altered diakinesis and metaphase I spermatocytes. Similarly, MCAK, that is recruited to WT meiotic centromeres in a SGO2-dependent manner (Gómez et al., 2007; Llano et al., 2008; Parra et al., 2009), isn’t loaded to the centromeres. The presence of a monopolar spindle together with the absence of the microtubule depolymerizing kinesin MCAK at the inner centromeres explains why bivalents aren’t able to align properly at the equatorial plate in altered metaphases I. Our data suggest that PLK1 is a key upstream regulator of the Bub1-H2AT120ph-SGO2 pathway during male mouse meiosis. This agrees with the fact that PLK1 associates with (Singh et al., 2021) and phosphorylates Bub1 (Qi et al., 2006; Grosstessner-Hain et al., 2011). All these findings support our suggestion that PLK1 could be directly phosphorylating and activating the kinase activity of Bub1, that in turn regulates the H2AT120ph phosphorylation and H2AT120ph-dependent loading of SGO2 and MCAK.

On the other hand, we have found that the inhibition of PLK1 doesn’t allow the phosphorylations of H3T3ph and Aurora B/C, and the loading of the CPC protein Borealin, at the inner centromere of altered diakinesis and metaphase I spermatocytes. It has been proposed that in mammalian somatic cells PLK1 phosphorylates and activates the kinase Haspin (Ghenoiu et al., 2013; Zhou et al., 2014). Thus, it is expected that by inhibiting PLK1, the kinase Haspin isn’t activated, and consequently, H3T3ph isn’t phosphorylated at the centromeres as we have found in spermatocytes. Our present results complement those we have recently reported after the chemical inhibition of the kinase Haspin on cultured seminiferous tubules, as well as in Haspin^−/−^ KO spermatocytes, indicating that in these situations H3T3ph isn’t phosphorylated at the inner centromere of metaphase I chromosomes (Berenguer et al., 2022). It is interesting to mention that we have found that PLK1 phosphorylated at serine S137 and T210 (PLK1S137ph and PLK1T210ph), both activated forms of PLK1 (Jang et al., 2002), are present at the inner centromere of WT metaphase I bivalents (Alfaro et al., 2021). Therefore, both PLK1 modifications are at the right place to regulate the phosphorylations of H2AT120ph, H3T3ph, and Aurora B/C, and the loading of SGO2, MCAK, and Borealin to the inner centromere of metaphase I bivalents.

In summary, this work presents data that support that PLK1 is a master regulator of male mouse meiosis progression *via* its involvement in the disassembly of LEs, and the assembly of the inner centromere domain.

## Materials and methods

### Mice

Seminiferous tubules from adult C57BL/6 wild-type (WT) male mice and REC8-*myc* transgenic male mice (Kudo et al., 2006) were used for this study.

### Organotypic culture of seminiferous tubules and inhibition of PLK1

The culture of seminiferous tubules from WT and REC8-*myc* mice was performed as previously described (Sato et al., 2011). Testes were removed, detunicated and fragments of seminiferous tubules were cultured for 2 h in agarose gel half-soaked in Minimum Essential Medium α culture medium (MEM α) (Gibco, A10490-01) supplemented with Knock Out Serum Replacement (KSR) (Gibco, 10828-010) and antibiotics (Penicillin/Streptomycin; Biochrom AG, A2213) at 34°C in an atmosphere with 5% CO_2_. The fragments of seminiferous tubules weren’t immersed in the medium, but rather deposited over the agarose gel absorbing the media from below, therefore requiring high concentrations when developing drug treatments (Alfaro et al., 2021). To inhibit PLK1, 100 µM BI 2536 (Selleck Chemicals, S1109) diluted in 10% DMSO was added to the culture medium, and seminiferous tubules were recovered after 8 h of treatment as we previously published (Alfaro et al., 2021). Controls were done with seminiferous tubules cultured with culture medium with added 10% DMSO.

### Indirect immunofluorescence

Seminiferous tubules were processed for squashing or chromatin spreading techniques as follows. For the squashing technique, portions of seminiferous tubules were collected from the culture and processed for indirect immunofluorescence as previously described (Page et al., 1998; Parra et al., 2002). Briefly, seminiferous tubules were fixed in freshly prepared 2% formaldehyde in PBS (137 mM NaCl, 2.7 mM KCl, 10.1 mM Na_2_HPO_4_, 1.7 mM KH_2_PO_4_, pH 7.4) containing .05% Triton X-100 (Sigma). After 10 min, several seminiferous tubules fragments were placed on a slide coated with 1 mg/ml poly-L-lysine (Sigma) with a small drop of fixative, and gently minced with tweezers. The tubules were then squashed, and the coverslip removed after freezing in liquid nitrogen. For the spreading technique, portions of seminiferous tubules were processed by the drying-down technique as previously described (Peters et al., 1997). For indirect immunofluorescence, slides of squashed or spreaded spermatocytes were rinsed three times for 5 min in PBS and incubated overnight at 4°C with primary antibodies diluted in PBS. Then, the slides were rinsed three times for 5 min in PBS and incubated for 1 h at room temperature with secondary antibodies. After other three rinsing steps, the slides were counterstained with 10 μg/ml 4′,6-diamidino-2-phenylindole (DAPI) for 3 min, rinsed in PBS for 1 min, mounted with Vectashield (Vector Laboratories) and sealed with nail polish.

### Antibodies

For indirect immunofluorescence the following primary antibodies were used at the indicated dilution in PBS: rabbit polyclonal anti-hSYCP3 (Abcam, ab-15092) at 1:100; mouse monoclonal anti-mSYCP3 (Santa Cruz, sc-74569) at 1:50; purified human anti-centromere autoantibody (ACA serum) revealing kinetochores (Antibodies Incorporated, 435-2RG-7) at 1:20; guinea-pig polyclonal anti-mHORMAD1 AB146, a gift of Attila Tóth (Wojtasz et al., 2009), at 1:50; rat monoclonal anti-α-Tubulin (Abcam, ab-6160) at 1:100; rabbit polyclonal anti-Pericentrin (Abcam, ab-4448) at 1:10; rabbit polyclonal anti-CENP-U phosphorylated at T78ph (Abcam, ab-34911) at 1:10; mouse monoclonal antibody against *myc* tag (GeneTex, GTX628259) at 1:20; rabbit polyclonal anti-H2AT120ph (Active Motif, 39,391) at 1:30; rabbit polyclonal anti-mSGO2 K1059, a gift of José Luis Barbero (Gómez et al., 2007) at 1:20; rabbit polyclonal anti-H3T3ph (Abcam, ab-17352) at 1:800; mouse monoclonal anti-hAurora A (T288ph)/Aurora B (T232ph)/Aurora C (T198ph), that we called Aurora Tph (Cell Signaling, 2914S) at 1:10; goat polyclonal against Lamin B (Santa Cruz, sc-6216) at 1:50; guinea-pig polyclonal anti-mSUN1, a gift of Manfred Alsheimer and Ricardo Benavente (Adelfalk et al., 2009) at 1:30, rabbit polyclonal anti-mREC8, a gift of Jibak Lee (Lee et al., 2003) at 1:10; rabbit polyclonal anti-RAD21L, a gift of Alberto Pendas (Herrán et al., 2011) at 1:10; rabbit polyclonal anti-RAD21, a gift of José Luis Barbero (Parra et al., 2004) at 1:10; sheep polyclonal anti-hMCAK, a gift of Linda Wordeman (Andrews et al., 2004) at 1:40; rabbit polyclonal anti-Borealin serum 1,647, a gift of William Earnshaw at 1:50; rabbit polyclonal anti-PLK1S137ph (Merk, 07-1348) at 1:10 (Du et al., 2015); and mouse monoclonal anti-PLK1T210ph (Abcam, ab-39068) at 1:10 (Du et al., 2015).

The secondary antibodies used were as follows: donkey anti-mouse conjugated with Alexa 488 (Molecular Probes, A-21202) or Alexa 594 (Molecular Probes, A-21203), donkey anti-rabbit conjugated with Alexa 488 (Molecular Probes, A-21206), goat anti-rabbit conjugated with Alexa 594 (Molecular Probes, A-11012), goat anti-human conjugated with Alexa 594 (Molecular Probes, A-11014), goat anti-guinea pig conjugated with Alexa 488 (Molecular Probes, A-11073), and donkey anti-sheep conjugated with FITC (Jackson ImmunoResearch, 713-095-147). All of them were employed at a 1:100 dilution in PBS.

### TUNEL assay

The DNA fragmentation-associated apoptosis of control and BI 2536-treated spermatocytes was detected by the TdT-mediated dUTP-fluorescein nick end labeling (TUNEL) assay by using a kit (Roche, 11684795910) according to manufacturer’s protocol. Nuclei were counterstained for 3 min with 10 μg/ml DAPI. Tests were developed on squashed 8 h control and 8 h BI 2536-treated seminiferous tubules. The percentage of apoptotic cells was calculated counting one thousand spermatocytes per condition.

### Image capture and processing

Immunofluorescence images were collected using an Olympus BX61 microscope equipped with epifluorescence optics, a motorized Z-drive and an Olympus digital camera (DP70 or DP71) controlled by analySIS software (Soft Imaging System). Figures presenting data obtained in squashed spermatocytes were obtained as image stacks and were processed to obtain complete Z-projections from 60–80 focal planes throughout the complete spermatocyte volume. Stacks were analyzed and processed, and in some cases three dimensional (3D) recontructions were made using the public domain software ImageJ (National Institutes of Health, United States; http://rsb.info.nih.gov/ij) for the generation of the supplementary videos. Final images were processed with Adobe Photoshop CS5 software.

## Data Availability

The raw data supporting the conclusion of this article will be made available by the authors, without undue reservation.

## Supplementary material

SUPPLEMENTARY FIGURE S1 Distribution of SYCP3 (green) and kinetochores (ACA, red) in diplotene (Dip.) , early diakinesis (E. dia.) **(C,D)**, and late diakinesis **(E,F)** WT squashed spermatocytes. Spermatocytes with unsubstracted **(A,C,E)** and substracted **(B,D,F)** SYCP3 nuclear background are shown. Numbers in each panel indicate the Z-projection of the indicated focal planes. White arrowheads indicate bulges and round SYCP3 thickenings along the desynapsed LEs, and yellow arrowheads indicate agglomerates at the nucleoplasm. Scale bar represents 2 μm.

SUPPLEMENTARY FIGURE S2 The breakdown of the nuclear envelope occurs in prometaphase I spermatocytes, and telomeres maintain their attachment to the nuclear envelope in diakinesis spermatocytes. Double immunolabelings of SYCP3 (red) with either Lamin B (green) or SUN1 (green), and counterstaining of the chromatin with DAPI (blue) on diplotene (Dip.) , early diakinesis (E. dia.) **(G)**, mid diakinesis (M. dia.) **(B)**, late diakinesis (L. dia.) **(C,H)**, prometaphase I (ProM I) **(D)** and metaphase I (M I) **(E)** WT squashed spermatocytes. Numbers in panels **(F–H)** indicate the Z-projection of different focal planes at top, equator and bottom regions of the spermatocytes and the complete projection. White arrowheads indicate bulges of SYCP3 along desynapsed LEs, whereas yellow arrowheads indicate SYCP3 agglomerates in the nucleoplasm or cytoplasm of diakinesis and metaphase I spermatocytes, respectively. Scale bar represents 5 μm.

SUPPLEMENTARY FIGURE S3 Distribution of SYCP3 in a representative metaphase I (M I) WT squashed spermatocyte. Immunolabeling of SYCP3 (green in the top row and pseudocolored in red in the bottom row), and counterstaining of the chromatin with DAPI. Numbers in each panel indicate the Z-projection of the indicated focal planes. Yellow arrowheads indicate SYCP3 agglomerates in the cytoplasm. Scale bar represents 2 μm.

SUPPLEMENTARY FIGURE S4 Distribution of SYCP3 and kinetochores during the diplotene/metaphase I transition. Double immunolabeling of SYCP3 (green) and kinetochores (ACA, red), and counterstaining of the chromatin with DAPI on WT squashed spermatocytes. Representative spermatocytes at mid diplotene (M. dip.), **(B)** early diakinesis (E. dia.), **(C)** mid diakinesis (M. dia.), **(D)** late diakinesis (L. dia.), **(E)** prometaphase I (ProM I), and **(F)** metaphase I (M I) spermatocytes are shown. **(A’–E’)** Enlarged autosomal bivalents at the corresponding prophase I stages. Enlarged autosomal **(G)** and sex **(H)** metaphase I bivalents. White arrowheads indicate elongated bulges and round thickenings of SYCP3 along desynapsed autosomal LEs. Yellow arrowheads indicate SYCP3 agglomerates in the nucleoplasm and cytoplasm of late diakinesis **(D)** and prometaphase I **(E)** spermatocytes. The sex chromosomes are indicated in **(H)**. Scale bars represent 5 μm in **(A–F)**, 2 μm in **(A’–E’,G)**, and 1 μm in **(H)**.

SUPPLEMENTARY FIGURE S5 Distribution of HORMAD1 and kinetochores during the late diplotene/metaphase I transition. Double immunolabeling of HORMAD1 (green) and kinetochores (ACA, red), and counterstaining of the chromatin with DAPI on WT squashed spermatocytes. Representative spermatocytes at late diplotene (L. dip.), **(B)** early diakinesis (E. dia.), **(C)** late diakinesis (L. dia.), **(D)** prometaphase I (ProM I), and **(E)** metaphase I (M I) spermatocytes are shown. **(F)** Enlarged autosomal metaphase I bivalent. White arrowheads indicate elongated bulges and round thickenings of HORMAD1 along asynapsed AEs of the sex chromosomes and desynapsed autosomal LEs. Yellow arrowheads indicate HORMAD1 agglomerates in the nucleoplasm and cytoplasm of late diakinesis **(C)**, prometaphase I **(D)** and metaphase I **(E)** spermatocytes. The sex bivalent is indicated in **(A)**. Scale bars represent 5 μm in **(A–E)**, and 2 μm in **(F)**.

SUPPLEMENTARY FIGURE S6 Distribution of SYCP3 in WT spread spermatocytes. Representative spermatocytes at early diplotene (E. dip.), **(C,D)** mid diplotene (M. dip.), **(E)** late diplotene (L. dip.), **(F)** early diakinesis (E. dia.), **(G)** mid diakinesis (M. dia.), **(H)** late diakinesis (L. dia.), **(I–K)** prometaphase I (ProM I), and **(L)** metaphase I (M I) WT spermatocytes are shown. The sex bivalent (XY) is indicated. Blue arrows denote the PAR region in the sex bivalent. White arrowheads indicate elongated bulges and thickenings along the asynapsed AE of the X chromosome and the desynapsed autosomal LEs. Yellow arrowheads denote agglomerates in the nucleoplasm of the late diakinesis nucleus **(H)** and cytoplasms of prometaphase I and metaphase I spermatocytes **(I–L)**. Scale bar represents 5 μm.

SUPPLEMENTARY FIGURE S7 Distribution of SYCP3 (green) in selected autosomal and sex bivalents from WT spread spermatocytes. Representative examples showing the morphological changes of the AEs/LEs in autosomal and **(B)** sex bivalents from diplotene up to metaphase I. Two autosomal bivalents, one of them with one interstitial and one distal chiasma **(A–F)**, and another one with a single interstitial chiasma **(G–L)**, are shown. White arrowheads indicate bulges and thickenings along the desynapsed autosomal LEs and asynapsed AEs of the X chromosome. Blue arrows denote the PAR region in the sex bivalent. Asterisks indicate the centromere region of the X chromosome. Yellow arrowheads denote agglomerates in the nucleoplasm and cytoplasms. Scale bar represents 1 μm.

SUPPLEMENTARY FIGURE S8 Distribution of REC8 and SYCP3 in early WT prophase I spermatocytes. Double immunolabeling of REC8-*myc* (green) and SYCP3 (red), and counterstaining of the chromatin with DAPI (blue) on squashed WT spermatocytes. Representative spermatocytes at leptotene (Lep.), **(B)** zygotene (Zyg.), and **(C)** pachytene (Pac.) stages are shown. White arrowheads indicate accumulations of SYCP3 at putative nucleoli. Scale bar represents 5 μm.

SUPPLEMENTARY FIGURE S9 Distribution of REC8 and SYCP3 in WT meiosis I spermatocytes. Double immunolabeling of REC8 (green) and kinetochores (ACA, red), and counterstaining of the chromatin with DAPI (blue) on squashed spermatocytes. Representative spermatocytes at diakinesis (Dia.) and **(B)** metaphase I (M I) are shown. The squared region in **(B)** appears enlarged. Scale bar represents 5 μm.

SUPPLEMENTARY FIGURE S10 TUNEL assay in 8 h control and BI 2536-treated **(B)** seminiferous tubules. TUNEL assay (red) and counterstaining of the chromatin with DAPI (blue). Unaltered and altered metaphases I denoted by white arrowheads are enlarged.

SUPPLEMENTARY FIGURE S11 Distribution of PLK1S137ph in WT spread spermatocytes. Double immunolabeling of PLK1S137ph (green) and SYCP3 (red), and counterstaining of the chromatin with DAPI (blue). Representative spermatocytes at leptotene (Lep.), **(B)** late zygotene (L. zyg.), **(C)** pachytene (Pac.), **(D)** early diplotene (E. dip.), **(E)** mid diplotene (M. dip), **(F)** late diplotene (L. dip.), **(J)** early diakinesis (E. dia.), **(K)** prometaphase I (ProM I), and **(L)** metaphase I are shown. **(G–I)** Selected late diplotene sex bivalents. Sex chromosomes (X, Y) and bivalents (XY) are indicated. White arrows point to centrosomes. Red arrowheads denote nuclear dense bodies. White arrowheads indicate elongated bulges and thickenings along the asynapsed AE of the X chromosome, and the desynapsed autosomal LEs. Scale bar represent 5 μm.

SUPPLEMENTARY FIGURE S12 Distribution of PLK1T210ph during the diplotene/metaphase I transition. Double immunolabeling of PLK1T210ph (green) and SYCP3 (red), and counterstaining of the chromatin with DAPI (blue). Representative spermatocytes at mid diplotene (M. dip), **(B)** late diplotene (L. dip.), **(C)** early diakinesis (E. dia.), **(D)** prometaphase I (ProM I), and **(E)** metaphase I are shown. Sex bivalents (XY) are indicated. White arrowheads indicate SYCP3 elongated bulges and thickenings along the asynapsed AE of the X chromosome, and the desynapsed autosomal LEs. Yellow arrowheads indicate SYCP3 agglomerates in the cytoplasm of prometaphase I and metaphase I **(D,E)** spermatocytes. Scale bar represent 5 μm.

SUPPLEMENTARY VIDEO S1 3D reconstruction of WT spermatocytes in diplotene, early diakinesis (Early dia.) and late diakinesis (Late dia.) after the immunolabeling of SYCP3 (green) and kinetochores (ACA, red) and chromatin counterstaining with DAPI (blue).

SUPPLEMENTARY VIDEO S2 3D reconstruction of control and BI 2536-treated diakinesis spermatocytes after the immunolabeling of SYCP3 (green) and kinetochores (ACA, red).

SUPPLEMENTARY VIDEO S3 3D reconstruction of control and BI 2536-treated metaphase I spermatocytes after the immunolabeling of SYCP3 (red) and kinetochores (ACA, red) and chromatin counterstaining with DAPI (blue).

SUPPLEMENTARY VIDEO S4 3D reconstruction of control and BI 2536-treated diakinesis spermatocytes after the immunolabeling of SYCP3 (red) and HORMAD1 (green) and chromatin counterstaining with DAPI (blue). The Z-projections of these spermatocytes are shown in Figures 5A,B.

SUPPLEMENTARY VIDEO S5 3D reconstruction of control and BI 2536-treated metaphase I spermatocytes after the immunolabeling of SYCP3 (red) and HORMAD1 (green) and chromatin counterstaining with DAPI (blue).

SUPPLEMENTARY VIDEO S6 3D reconstruction of control and BI 2536-treated diakinesis spermatocytes after the immunolabeling of SYCP3 (red) and REC8-*myc* (green) and chromatin counterstaining with DAPI (blue).

SUPPLEMENTARY VIDEO S7 3D reconstruction of control and BI 2536-treated metaphase I spermatocytes after the immunolabeling of SYCP3 (red) and REC8-*myc* (green) and chromatin counterstaining with DAPI (blue).

## Abbreviations

AEs, axial elements; CE, central element; LEs, lateral elements; SC, synaptonemal complex.
