# Supplementary figures and images for: Kinase PLK1 regulates the disassembly of the lateral elements and the assembly of the inner centromere during the diakinesis/metaphase I transition in male mouse meiosis
Source: Front Cell Dev Biol. 2023 Jan 13;10:1069946. doi: 10.3389/fcell.2022.1069946 (PMC9887526; doi:10.3389/fcell.2022.1069946)

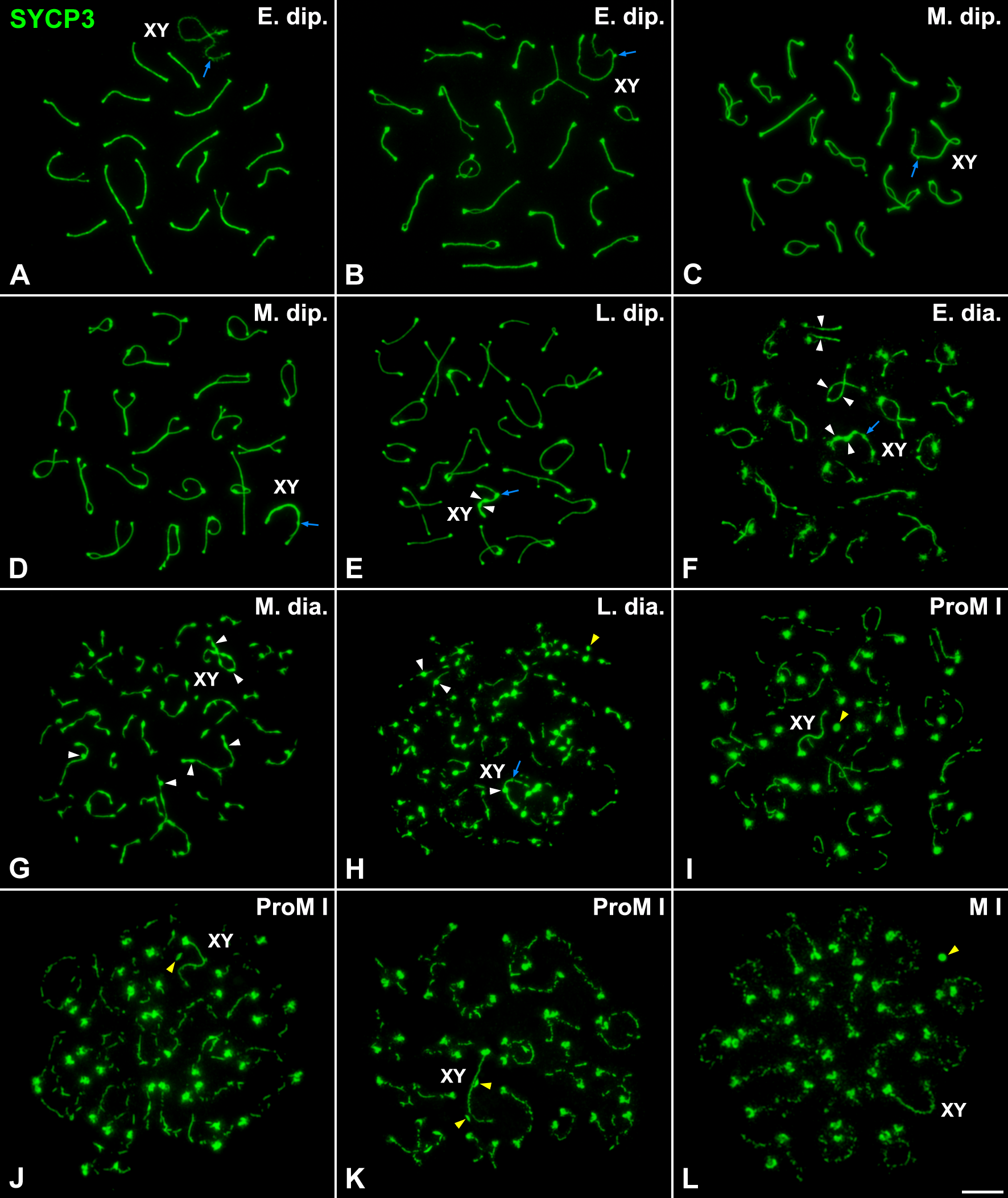

Supplement: Supplementary file 3 [file Image6.TIF]

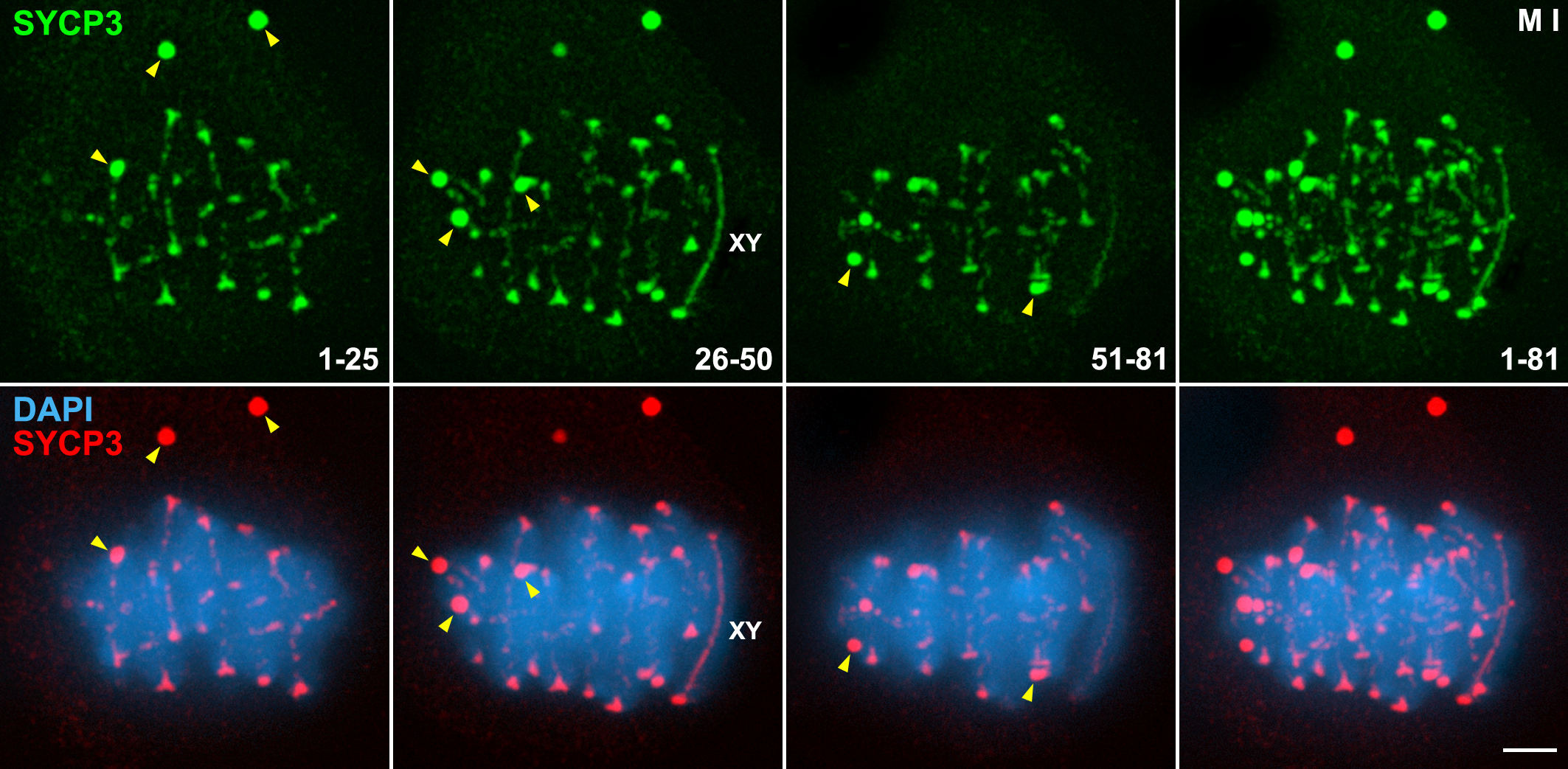

Supplement: Supplementary file 4 [file Image3.TIF]

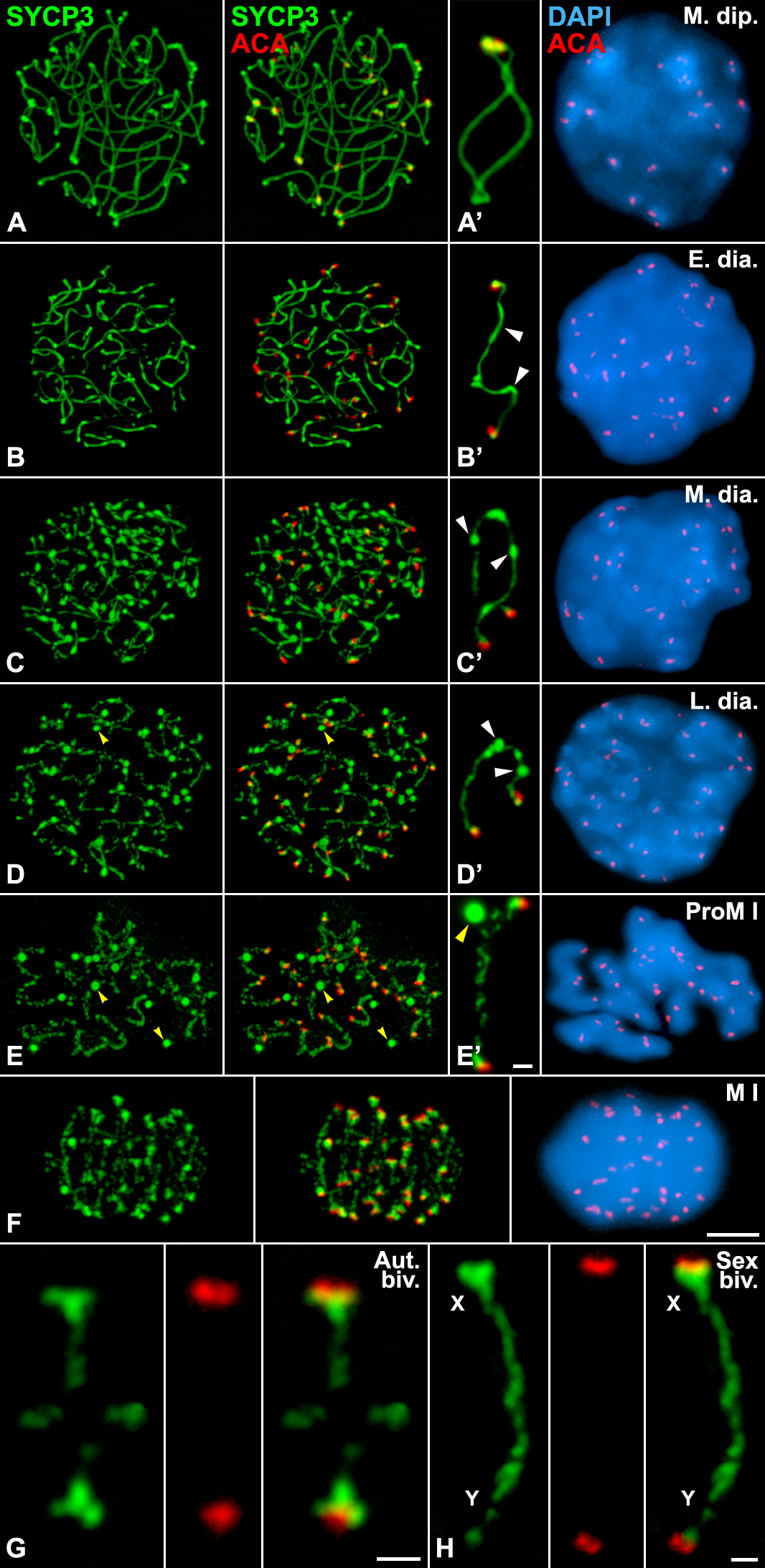

Supplement: Supplementary file 5 [file Image4.TIF]

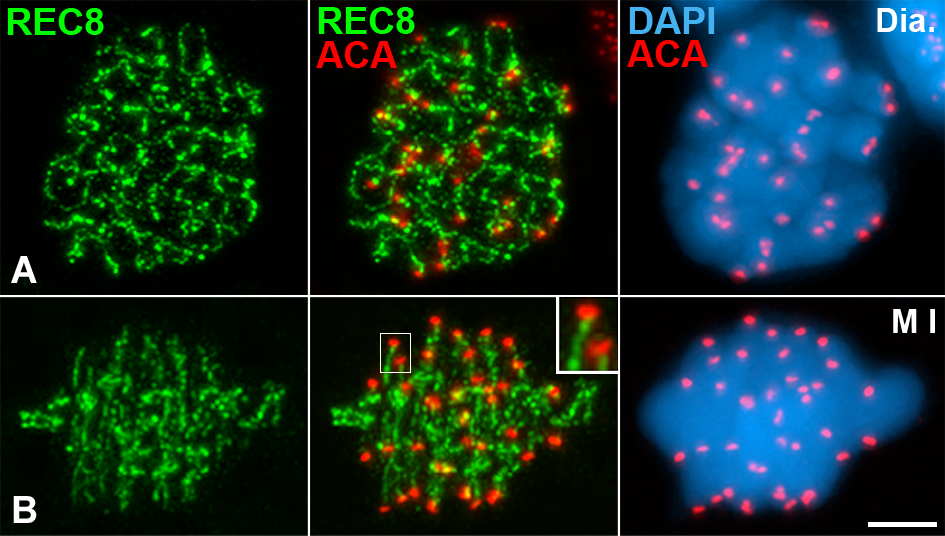

Supplement: Supplementary file 6 [file Image9.TIF]

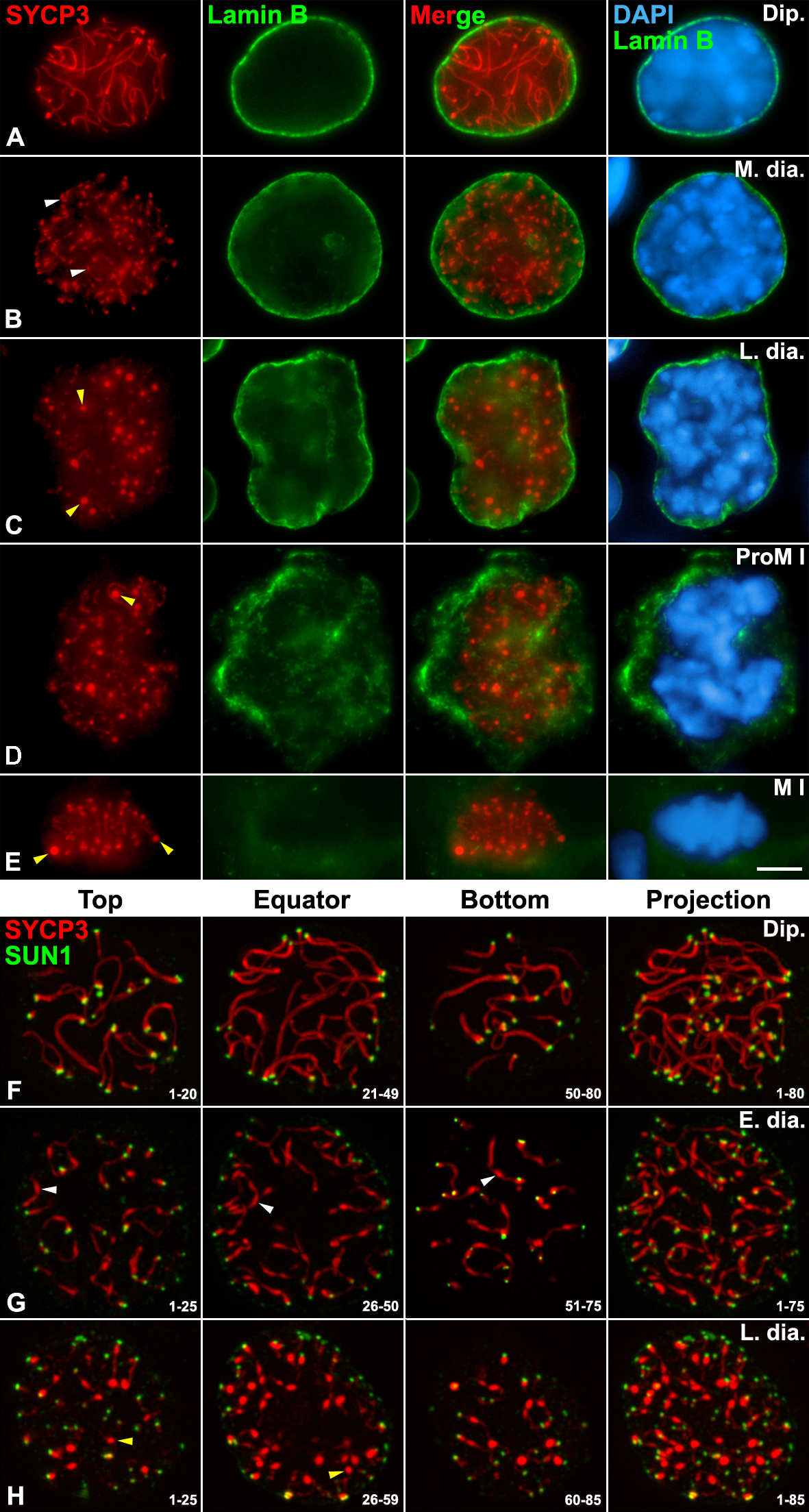

Supplement: Supplementary file 7 [file Image2.tif]

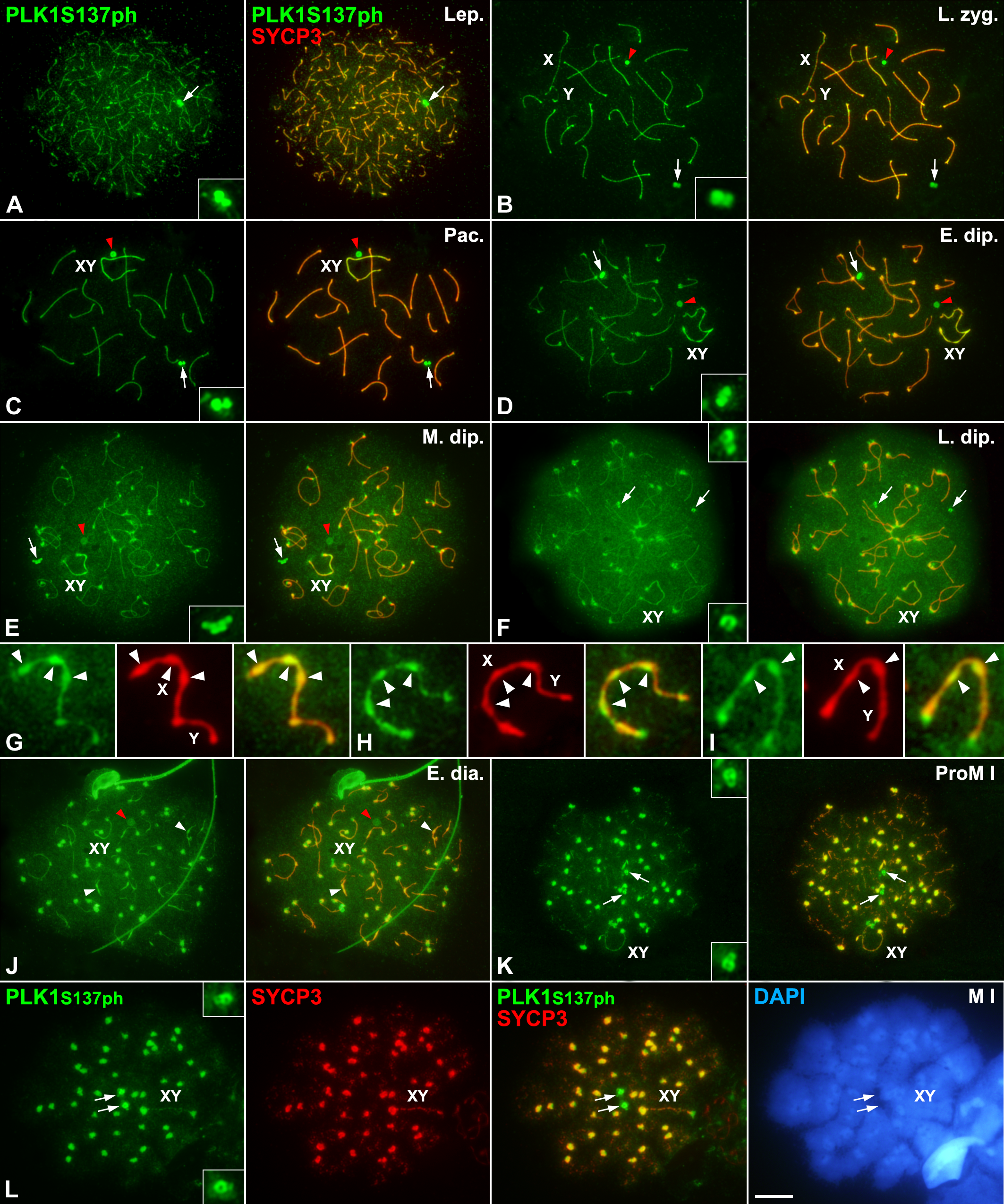

Supplement: Supplementary file 8 [file Image11.TIF]

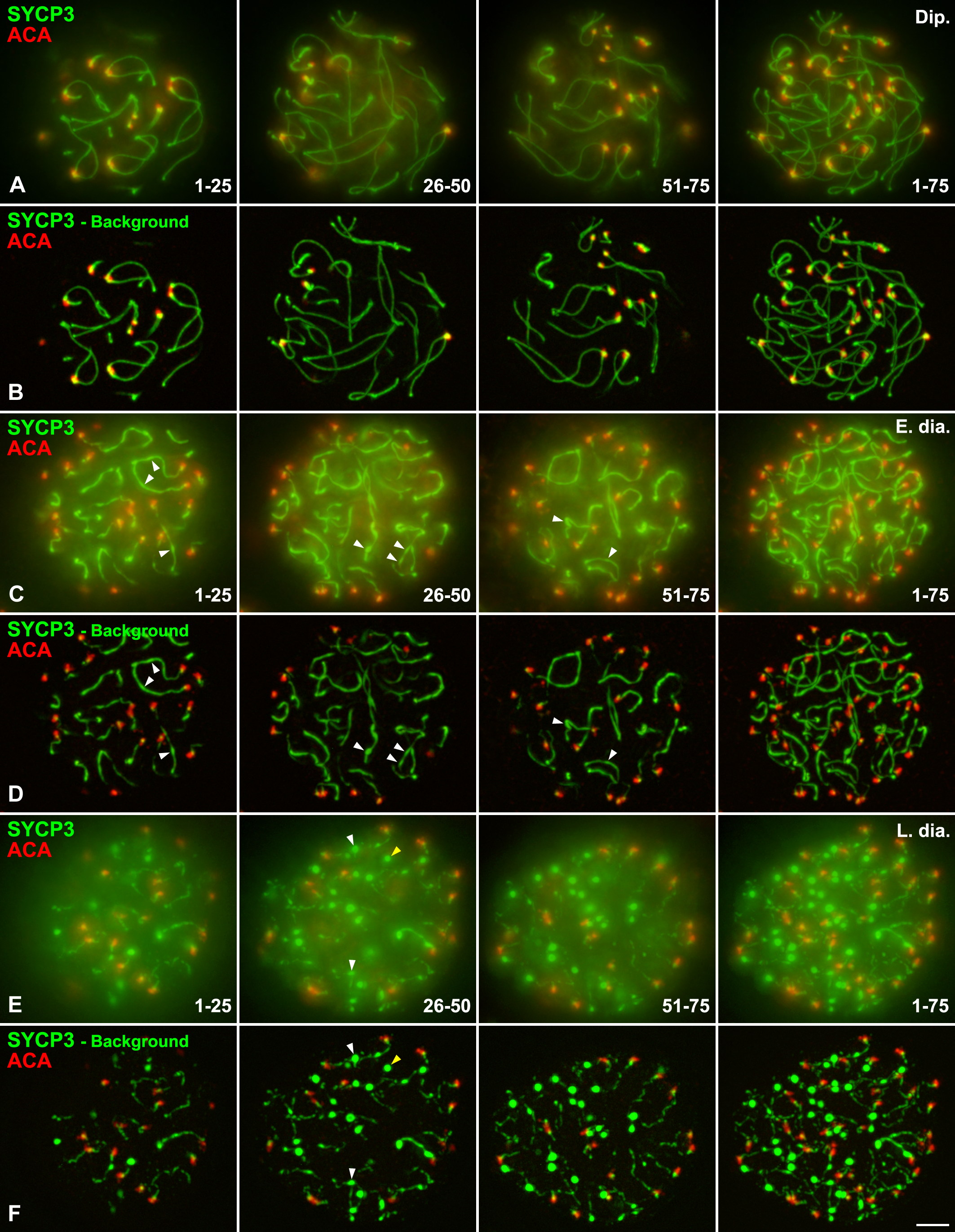

Supplement: Supplementary file 9 [file Image1.TIF]

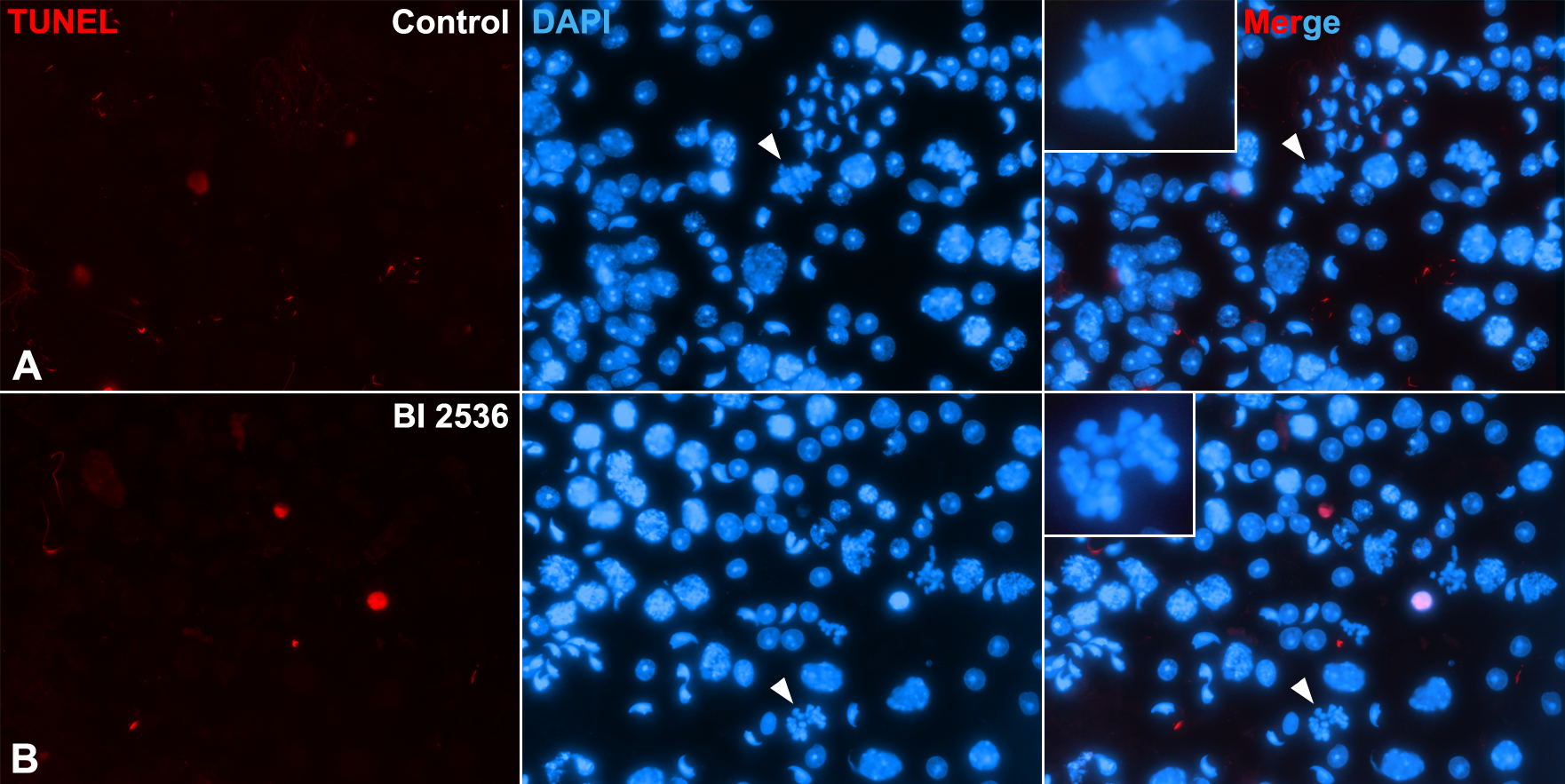

Supplement: Supplementary file 10 [file Image10.TIF]

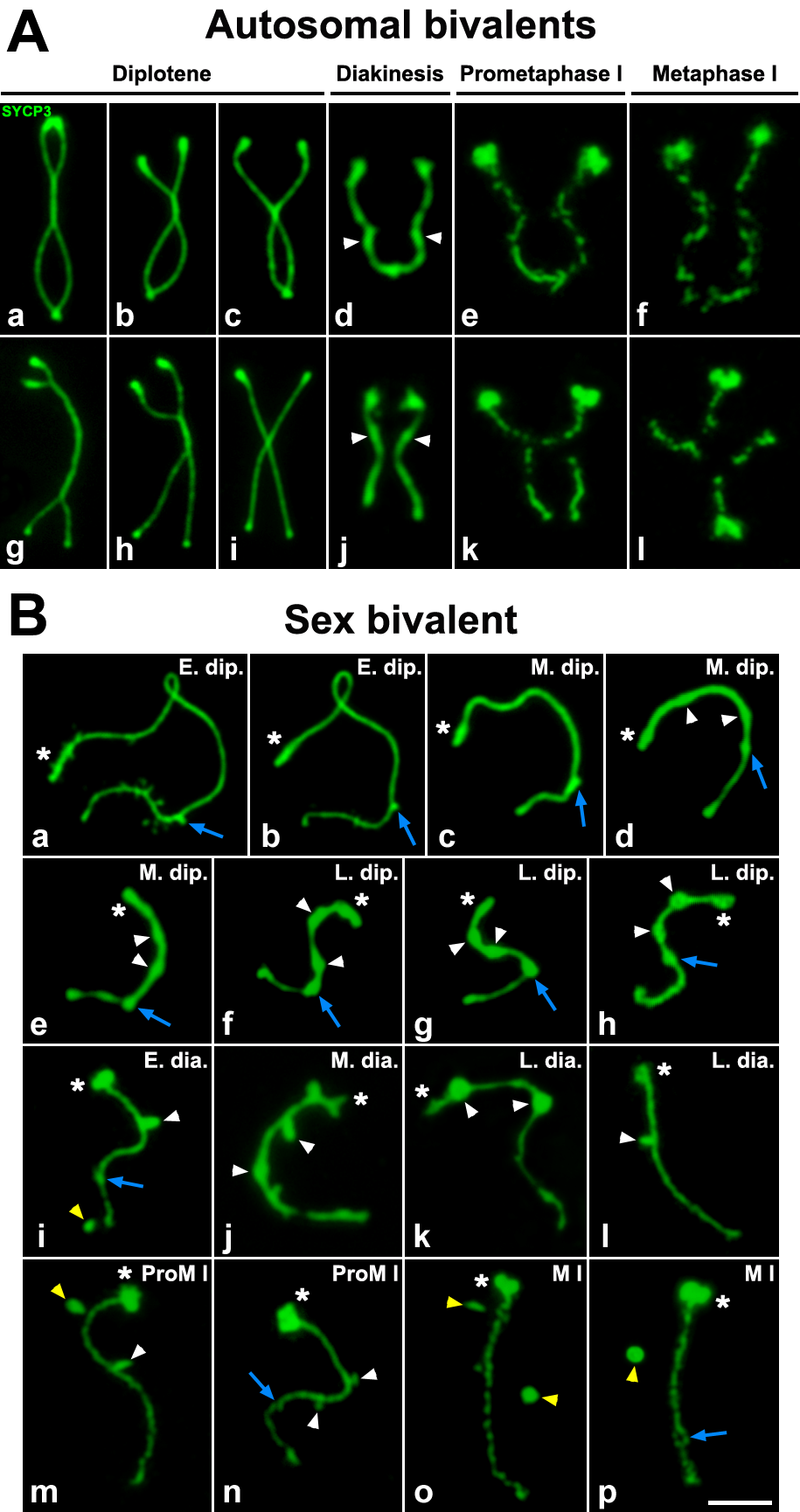

Supplement: Supplementary file 11 [file Image7.TIF]

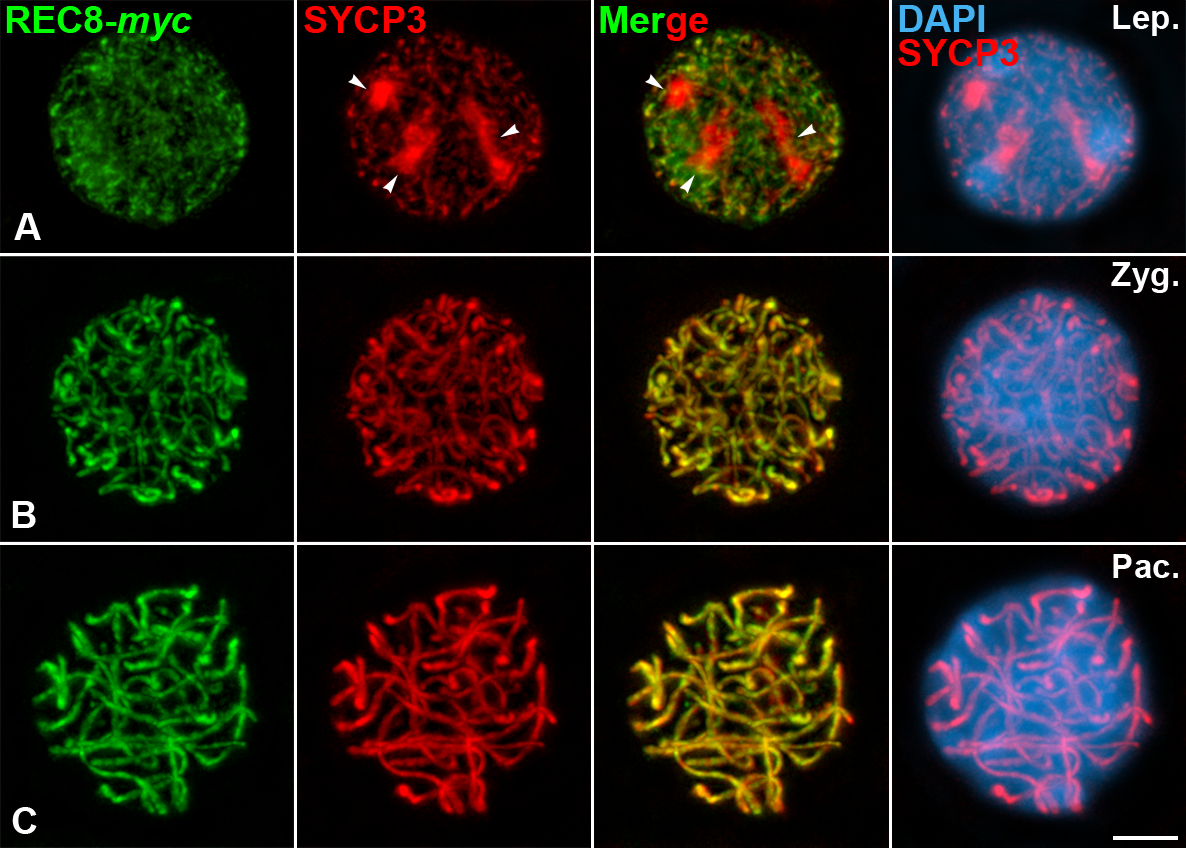

Supplement: Supplementary file 13 [file Image8.TIF]

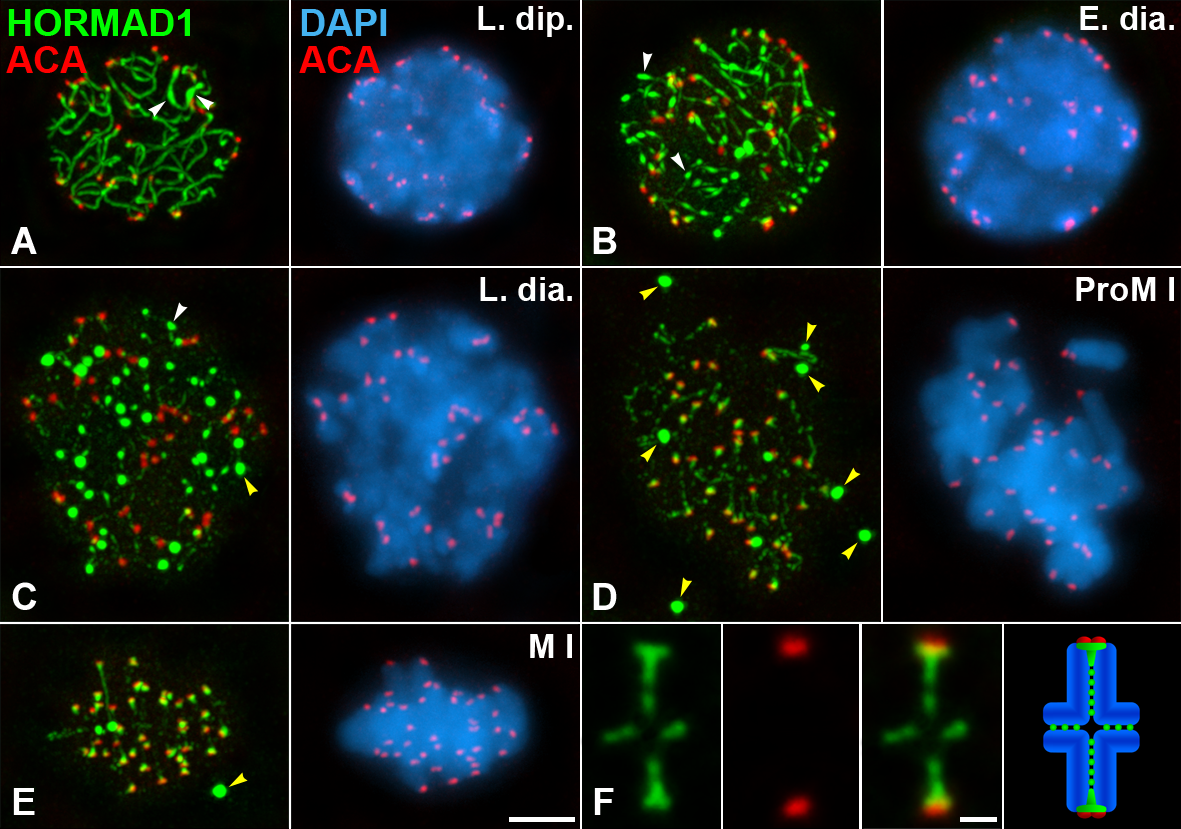

Supplement: Supplementary file 14 [file Image5.TIF]

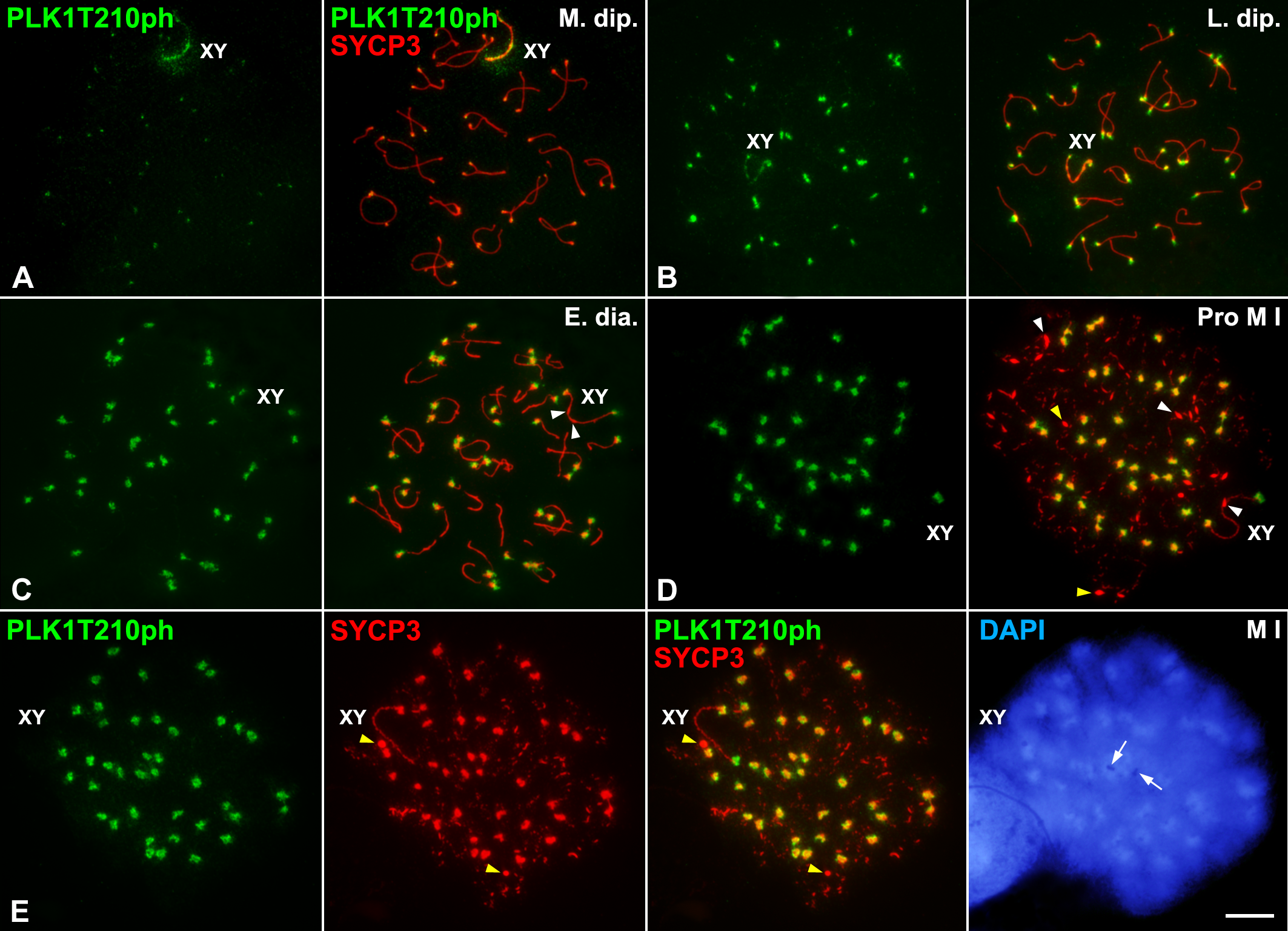

Supplement: Supplementary file 18 [file Image12.TIF]
